# Time-optimized protein NMR assignment with an integrative deep learning approach using AlphaFold and chemical shift prediction

**DOI:** 10.1126/sciadv.adi9323

**Published:** 2023-11-22

**Authors:** Piotr Klukowski, Roland Riek, Peter Güntert

**Affiliations:** ^1^ Institute of Molecular Physical Science, ETH Zurich, Vladimir-Prelog-Weg 2, 8093 Zurich, Switzerland.; ^2^ Institute of Biophysical Chemistry, Goethe University Frankfurt, Max-von-Laue-Str. 9, 60438 Frankfurt am Main, Germany.; ^3^ Department of Chemistry, Tokyo Metropolitan University, 1-1 Minami-Osawa, Hachioji, 192-0397 Tokyo, Japan.

## Abstract

Chemical shift assignment is vital for nuclear magnetic resonance (NMR)–based studies of protein structures, dynamics, and interactions, providing crucial atomic-level insight. However, obtaining chemical shift assignments is labor intensive and requires extensive measurement time. To address this limitation, we previously proposed ARTINA, a deep learning method for automatic assignment of two-dimensional (2D)–4D NMR spectra. Here, we present an integrative approach that combines ARTINA with AlphaFold and UCBShift, enabling chemical shift assignment with reduced experimental data, increased accuracy, and enhanced robustness for larger systems, as presented in a comprehensive study with more than 5000 automated assignment calculations on 89 proteins. We demonstrate that five 3D spectra yield more accurate assignments (92.59%) than pure ARTINA runs using all experimentally available NMR data (on average 10 3D spectra per protein, 91.37%), considerably reducing the required measurement time. We also showcase automated assignments of only ^15^N-labeled samples, and report improved assignment accuracy in larger synthetic systems of up to 500 residues.

## Teaser

Automated protein NMR assignments by a deep learning approach that exploits AlphaFold structures to shortens NMR measurements.

## INTRODUCTION

Nuclear magnetic resonance (NMR) spectroscopy is a key analytical technique that provides detailed information on the structure, dynamics, and interactions of proteins. These data can be obtained simultaneously for a large number of individual atom positions using the intrinsically present probes of nuclear spins. To achieve this atomic resolution, it is necessary to attribute resonance frequencies of nuclear spins, expressed as chemical shifts, to individual atoms in the protein (*1*). This chemical shift assignment is a key task in most NMR studies of proteins. It is generally achieved by recording and analyzing a set of multidimensional NMR spectra. Each cross peak in an *n*-dimensional spectrum correlates *n* atoms with each other, and alignments among the cross peaks make it possible to uniquely link chemical shift values to individual atoms in the chemical structure of the protein. This process is generally demanding in terms of NMR measurements and spectra analysis. Most of the spectrometer measurement time in a biomolecular NMR project is frequently spent on measuring spectra for the chemical shift assignment, which are not of direct use to the biological question at stake, such as, for instance, elucidating dynamics or interactions of the protein. The same holds for the time spent by the spectroscopist: Finding chemical shift assignments is time consuming and requires expertise.

To change this situation by accelerating NMR chemical shift assignment, one should reduce the number of spectra required and automate their analysis without compromising the reliability of the results. Here, we present a method that achieves this by exploiting recent advances in machine learning and by efficiently incorporating the information contained in three-dimensional (3D) protein structures into the assignment process. The latter serve to replace information that would otherwise have to be gathered from additional NMR spectra.

Given the central importance of chemical shift assignments, many approaches have been proposed to automate their determination (*2*, *3*). They differ regarding input data (e.g., spectra, peak lists, spin systems, and additional data), algorithms, and output [e.g., backbone (*4*), side chain (*5*–*7*), or methyl group assignments (*8*)]. A general automated assignment method that can be applied in all these cases is FLYA (*7*), implemented in the Combined assignment and dYnamics Algorithm for NMR Applications software package (CYANA) (*9*, *10*). It uses an evolutionary algorithm to find an optimal mapping between the cross peaks that are expected for a given protein and set of spectra and the cross peaks identified in the corresponding experimentally measured spectra. FLYA has recently been embedded in the ARTificial Intelligence for NMR Applications method (ARTINA) (*11*) and the NMRtist webserver (*12*) that use machine learning for visual spectrum analysis and other tasks to automate the entire process of protein NMR data analysis from raw spectra to assignments and the 3D structure. Using a large dataset of 1329 experimental solution NMR spectra for 100 monomeric globular proteins, it was shown that ARTINA is able to assign correctly 91.4% of all backbone and side-chain chemical shifts for which reference assignments are known (*11*). ARTINA thus reduces the effort for the chemical shift assignment of a protein assignment essentially to the preparation of the sample and the spectra measurements.

However, on average, more than 13 multidimensional NMR spectra were used to obtain these results, which amounts to more than 2 weeks of NMR measurement time per protein using traditional acquisition schemes. Considering that the computation time of the ARTINA algorithm is typically less than 2 hours and that the operation of an NMR spectrometer is much more costly and demanding than that of a computer, reducing the number of spectra used for the assignment is an obvious strategy to progress in the efficiency of biomolecular NMR projects.

Knowledge of the 3D structure of a protein can support the chemical shift assignment in mainly two ways (*13*–*15*): by more realistic prediction of the expected cross peaks in nuclear Overhauser effect spectroscopy (NOESY) spectra and through structure-based predictions of chemical shift values. This has become particularly relevant because AlphaFold (*16*) can now accurately predict the 3D structure of most structured proteins. We have therefore built an integrative machine learning–based method for structure-based NMR chemical shift assignment. Here, we evaluate its performance and identify optimal sets of spectra for the assignment of backbone amide groups or of all chemical shifts.

The structure of this paper is as follows. Starting from the 1170 experimental spectra for 89 proteins of the original publication on ARTINA (*11*), we define 25 different sets of input spectra for automated assignment calculations with ARTINA. In the first part of the paper, the accuracy of chemical shift assignments is evaluated in three different scenarios: assignment of backbone amide groups by “classical” triple-resonance assignment spectra, assignment of backbone amide groups by 3D NOESY and triple-resonance spectra, and complete assignment of backbone and side-chain chemical shifts, comparing in each case the assignment results obtained by ARTINA either without structural input, or using the structure only for the generation of expected NOESY cross peaks, or using the structure in addition for the prediction of chemical shifts with the UCBShift method (*17*). In addition, we tested the feasibility of backbone amide assignment using NMR spectra recorded with only ^15^N-labeled samples, which could constitute an alternative to costly double labeling with ^13^C/^15^N. In the second part of the paper, we evaluate the impact of the accuracy of the input structures on the assignments using a large number of well-folded and well-packed decoys generated by 3DRobot (*18*) that deviate by 0- to 5-Å root mean square deviation (RMSD) from the experimental structure. In the third part of the paper, we assess how the integrative approach performs for the assignment of large synthetically generated protein systems (up to 500 residues), which are currently rarely deposited in the Biological Magnetic Resonance Data Bank (BMRB) database. In conclusion, together with our integrative approach and its evaluation, we propose a set of data-driven practical recommendations for performing chemical shift assignments of proteins.

## RESULTS

### Experimental and derived data

To evaluate the effectiveness of various automated assignment strategies incorporating ARTINA, AlphaFold, and UCBShift, we used 1170 2D, 3D, and 4D NMR spectra that allow to reproduce the assignments of 89 proteins directly out of the original measurements. This dataset has previously been used for both the manual (BMRB depositions) and automated (*11*) assignments of these proteins. We used these spectra to form 25 benchmark datasets (Table 1), which vary in the number of spectra used as input for the assignment. In addition to the complete set of spectra (dataset 1), 24 other datasets comprising one to eight specific 3D spectra per protein were formed. Datasets 1 to 17 are suitable for complete (backbone and side-chain) assignment, whereas datasets 18 to 25 are designed for backbone amide assignment. The last dataset (25), comprising only [^1^H,^15^N]-HSQC and ^15^N-edited [^1^H,^1^H]-NOESY, has been designed to evaluate the performance of the method with ^15^N-labeled samples. In addition to 3D spectra, all sets include one or two 2D spectra, [^1^H,^15^N]-HSQC and [^1^H,^13^C]-HSQC. NOESY spectra were included in most of the sets because they have a high information content, work well also for larger proteins, and provide distance information, which is useful beyond chemical shift assignment, e.g., for structure determination or multistate structure determination with eNOEs (*19*).

**Table 1.** Overview of the 25 spectra sets used to assess the performance of automated assignment strategies that incorporate ARTINA, AlphaFold, and UCBShift. Each dataset features a unique combination of multidimensional NMR spectra that serve as the input for assignments. Spectra are denoted by ■ in datasets for complete (backbone and side-chain) assignment, by ● in datasets suitable only for backbone amide assignment, and by ◆ in the dataset requiring only ^15^N labeling.

Dataset ID	1	2	3	4	5	6	7	8	9	10	11	12	13	14	15	16	17	18	19	20	21	22	23	24	25
Number of 3D spectra used*	9.8	6	5	6	8	5	4	5	7	4	5	6	3	3	4	2	2	3	3	2	2	2	1	2	1
Proteins with these spectra†	89	31	41	25	23	42	54	32	26	44	30	26	51	68	34	77	84	30	41	34	44	44	51	75	84
[^1^H,^13^C]-HSQC	■	■	■	■	■	■	■	■	■	■	■	■	■	■	■	■									
[^1^H,^15^N]-HSQC	■	■	■	■	■	■	■	■	■	■	■	■	■	■	■	■	■	●	●	●	●	●	●	●	◆
^13^C-edited [^1^H,^1^H]-NOESY	■	■	■	■	■	■	■	■	■	■	■	■	■	■	■	■	■								
^15^N-edited [^1^H,^1^H]-NOESY	■	■	■	■	■	■	■	■	■	■	■	■	■	■	■	■	■							●	◆
HNCA	■	■		■	■	■		■	■	■	■	■	■		■			●	●	●	●	●	●		
hNcoCA	■			■	■			■	■		■	■			■			●		●					
HNCO	■				■				■		■	■						●							
hNcaCO	■																								
CBCANH	■				■				■			■							●			●			
CBCAcoNH	■	■	■			■	■			■				■					●		●			●	
HBHAcoNH	■																								
HCCHTOCSY	■	■	■	■	■	■	■	■	■																
hCcoNH	■																								
CCHTOCSY	■	■	■	■	■																				
All others	■																								

*Number of 3D spectra included in the dataset. Dataset 1 includes all experimental spectra available for a given protein (*11*). The number of 3D spectra given for dataset 1 is the average over all proteins. All other datasets use the given, fixed number of spectra. For certain proteins, ^13^C-edited [^1^H,^1^H]-NOESY was recorded separately for aliphatic and aromatic ^13^C. These are counted as a single 3D spectrum per protein.

†Number of proteins for which all spectra of the specified types are available. While this study used 89 proteins, the availability of different types of spectra varies among proteins, resulting in a variable number of proteins within each dataset. For instance, in dataset 16, only 77 proteins have both ^13^C- and ^15^N-edited NOESY in conjunction with both HSQC experiments.

In addition to NMR spectra, we collected several other types of data for each protein under study: (i) a manually solved protein structure [the Protein Data Bank (PDB) deposition, if available], (ii) a manually assigned list of chemical shifts (BMRB deposition), (iii) an AlphaFold structure prediction (five models), (iv) 100 decoy structures, and (v) a UCBShift chemical shift list prediction.

We generated AlphaFold structure predictions using only PDB templates that were deposited in the data bank before the publication of the query protein, using as input the sequence of the protein construct for which the NMR spectra had been recorded. To prepare the input for the assignment calculations presented here, we combined the five structure candidates that are predicted by AlphaFold using default parameters into a structure bundle that resembles NMR models. This approach was overall more advantageous for automated chemical shift assignment than using a single, “best” AlphaFold prediction because it leads to a more realistic generation of expected NOESY peaks in particular for not well-defined parts of the structure (e.g., surface side chains, flexible chain ends, and loops). These are not straightforward to detect from the coordinates of a single structure but often lead to diverging conformers in a structure bundle, from which fewer spurious expected NOESY cross peaks are generated by requiring the corresponding distance to be short in all conformers simultaneously. When a single AlphaFold structure is used, the number of back-calculated through-space contacts is substantially (by 32 and 23% for the ^13^C- and ^15^N-edited NOESY, respectively) higher than the number of contacts extracted from the AlphaFold structure bundle. These surplus contacts do not contribute positively to the combinatorial optimization used in chemical shift assignment (*7*). Throughout experiments with our method, we observed that adopting a conservative strategy—taking only the set of contacts observed in all AlphaFold predicted model proposals—yields higher accuracy. This observation aligns with the design of the FLYA algorithm (*7*), which strives to assign all expected NOESY contacts. If some of them are present in only one AlphaFold structure (and absent in others), the probability that the corresponding signal is visible in the experimental spectra is low.

In addition, we used 3DRobot (*18*) to create well-structured decoys for each benchmark protein used in this study. The decoy structures, refined at the atomic level through energy optimization, bear similarities with the true folds deposited in the PDB, such as the number and placement of secondary structure elements (figs. S1 and S2). Each AlphaFold (or decoy) structure served also as input for chemical shift prediction using UCBShift (*17*), yielding C^α^ C^β^, C′, H^N^, H^α^, and N shift predictions.

To ensure integrity of our benchmark data, we implemented rigorous cross-verification across all prepared data modalities. Manually determined chemical shift lists (from BMRB) and protein structures (from PDB, if available) were used to back-calculate expected cross peak positions. These positions were subsequently overlaid onto contour plots of the spectra (1170 2D, 3D, and 4D experiments), ensuring no systematic shifts or referencing inconsistencies existed between depositions in public repositories and recorded experiments. Following this, we compared AlphaFold-generated protein structure bundles with manually derived structures, as indicated in table S1. The results revealed good agreement, with an average backbone RMSD of 1.03 Å. There are only four proteins in the benchmark dataset for which the AlphaFold structure prediction error exceeded 2 Å: 2LX7, 6FIP, 2LVN, and 2LND. Furthermore, we compared UCBShift predictions (using AlphaFold structures as input) against BMRB depositions, finding that the errors are comparable to those reported in the literature (*17*): C′ (RMSD of 0.85 ppm versus 0.81 ppm reported), C^α^ (0.77 ppm/0.81 ppm), C^β^ (0.86 ppm/1.00 ppm), H^N^ (0.39 ppm/0.31 ppm), H^α^ (0.20 ppm/0.19 ppm), and N (1.98 ppm/1.81 ppm). Last, we manually inspected the decoy structures and compared them with their corresponding true folds, as illustrated in figs. S1 and S2. This multilayered validation process was instrumental in reinforcing the robustness of our data preparation methods.

### Impact of experimental data quantity on assignment accuracy

**Fig. 1.**
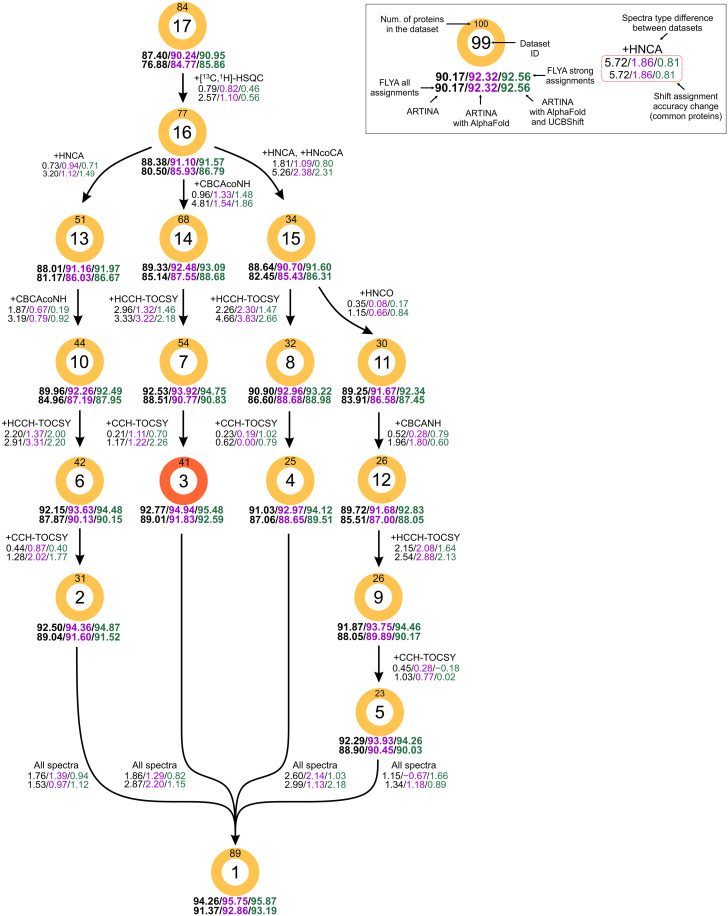
Impact of experimental data quantity on the accuracy of complete protein chemical shift assignment. Each node in the graph represents a single dataset specified in Table 1, with edges connecting datasets with similar spectrum types and highlighting the differences between them (e.g., the arrow from dataset 16 to 13 corresponds to the addition of the HNCA spectrum). Beneath each node, the chemical shift assignment accuracy (defined in Table 2) is reported for three independent runs: ARTINA only (black), ARTINA with AlphaFold structure (magenta), and ARTINA with AlphaFold and UCBShift predictions (green). As subsets contain variable numbers of proteins (as indicated in Table 1), we report the relative change in chemical shift assignment accuracy next to each edge in the graph, calculated using the proteins available in both datasets connected by the edge. Nodes are sorted from top to the bottom, beginning with datasets containing the least number of spectra. As the graph is traversed from top to bottom, the performance of the method with complementary inputs (green) begins to surpass the performance of the ARTINA-only approach with the full input dataset (dataset 1, black), such as in the recommended dataset 3 (highlighted in red).

**Fig. 2.**
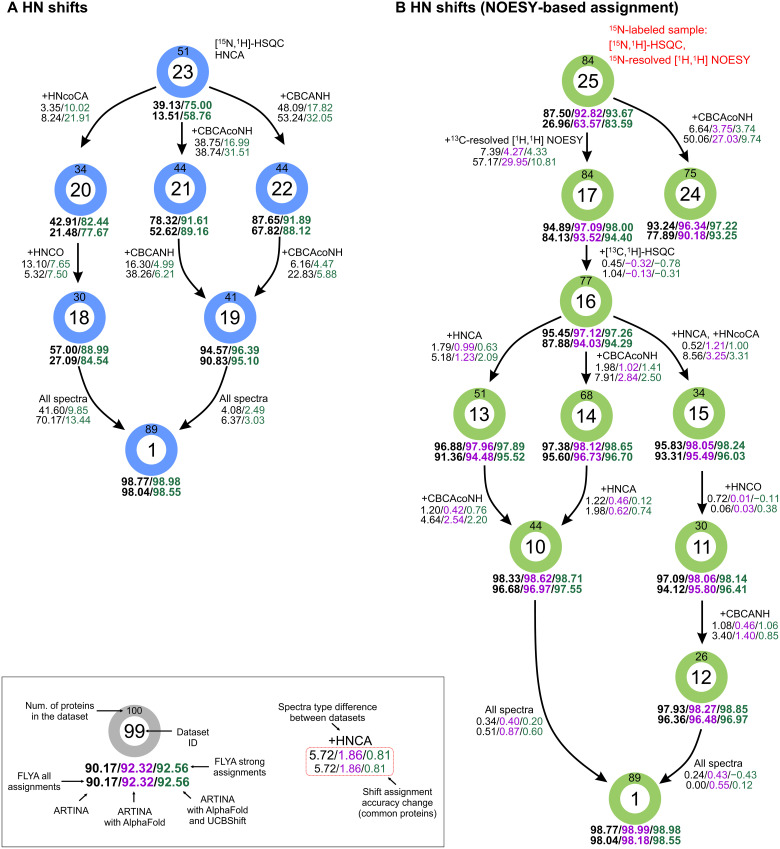
Impact of experimental data quantity on the accuracy of backbone amide chemical shift assignment. (**A**) Assignment using through-bond triple-resonance backbone assignment spectra. (**B**) NOESY-based assignment, optionally including triple-resonance experiments. The graphs are structured as in Fig. 1. Each node in the graphs represents a single dataset specified in Table 1, with edges connecting datasets with similar spectrum types and highlighting the differences between them [e.g., the arrow from dataset 23 to 22 in (A) corresponds to adding the CBCANH spectrum]. Beneath each node, the chemical shift assignment accuracy is reported for three independent runs: ARTINA only (black), ARTINA with AlphaFold structure (magenta, only for NOESY-based assignment), and ARTINA with AlphaFold and UCBShift predictions (green). As subsets contain variable numbers of proteins (as indicated in Table 1), we report the relative change in chemical shift assignment accuracy next to each edge in the graph, calculated using proteins available in both datasets connected by the edge. Nodes are sorted from top to the bottom, beginning with datasets containing the least input data. As the graph is traversed from top to bottom, the performance of the method with complementary inputs (green) begins to surpass the performance of ARTINA-only approach with full input dataset (dataset 1, black).

Automated chemical shift assignment calculations were performed with the datasets specified in Table 1 and either no additional input (ARTINA), using AlphaFold structures as input for expected NOESY cross peak generation (ARTINA with AlphaFold), or using AlphaFold structures as input for expected NOESY cross peak generation as well as for chemical shift prediction with UCBShift (ARTINA with AlphaFold and UCBShift). To conveniently present the impact of different spectrum types on the assignment, we arranged the datasets in the graphs shown in Figs. 1 and 2. Each path in the graphs proceeds from an initial minimal spectrum set by gradually increasing the number of spectra toward the common end point, i.e., the full dataset. The most important findings are summarized in Table 2. Distributions of the chemical shift assignment accuracy are shown for selected spectra sets in fig. S4. The relation between chemical shift assignment accuracy and protein size is visualized in fig. S5.

**Table 2.** Summary of key assignment results. The table consolidates key outcomes from Figs. 1 and 2, evaluating the integrative approach across two tasks—full and backbone amide group assignments—using varying quantities of input data. Rows in bold correspond to the recommended assignment strategies, as presented in Figs. 1 and 2.

Spectra set (dataset ID)*	Number of 3D spectra	Assignments (%)†				
		Correct (strong)	Completeness (strong)	Strong	Correct (weak)	Correct (all)
						
*Full backbone and side-chain assignment:*						
All (dataset 1), without structure	9.8	94.26	88.67	94.40	44.44	91.37
All (dataset 1)	9.8	95.87	89.69	94.08	52.87	93.19
**Optimal (dataset 3)**	**5**	**95.48**	**89.79**	**93.73**	**52.00**	**92.59**
Optimal, without CCHTOCSY (dataset 7)	4	94.75	86.66	92.23	46.72	90.84
NOESY-only (dataset 16)	2	90.95	78.99	87.75	46.15	85.86
*Backbone amide group assignment:*‡						
All (dataset 1), without structure	13	98.77	97.78	99.24	22.22	98.04
All (dataset 1)	13	98.98	97.83	98.77	25.00	98.55
Optimal (dataset 3)	5	98.85	95.71	97.55	33.33	96.36
**HNCA, CBCAcoNH, CBCANH (dataset 19)**	**3**	**96.39**	**94.12**	**98.89**	**0.00**	**95.10**
**NOESY-only (dataset 17)**	**2**	**98.00**	**92.31**	**93.97**	**33.33**	**94.40**
^15^N-NOESY-only (dataset 25)	1	93.67	78.06	82.88	31.37	83.59

*Datasets are defined in Table 1. Their IDs are given in parenthesis. Dataset “all” includes all experimental spectra available for a given protein. The “optimal” set comprises ^13^C- and ^15^N-edited [^1^H-^1^H]-NOESY, CBCAcoNH, HCCHTOCSY, and CCHTOCSY spectra. In addition, 2D [^1^H,^15^N]-HSQC and [^1^H,^13^C]-HSQC were used in all cases. Unless noted otherwise, 3D structures predicted by AlphaFold were used for the generation of expected NOESY peaks and chemical shift prediction.

†Percentages of assignments are defined as follows. Let *N* and $\bar{N}$ be the number of atoms for which the assignment by ARTINA and the reference assignment from the BMRB deposition agree/disagree within a tolerance of 0.03 ppm for ^1^H and 0.4 ppm for ^13^C and ^15^N. Only atoms assigned by both methods can contribute to *N* or $\bar{N}$. These quantities are calculated for the assignments that are classified by FLYA as strong (*N*_strong_ and $\bar{N}$_strong_), weak (*N*_weak_ and $\bar{N}$_weak_), or in either of the two classes (*N*_all_ = *N*_strong_ + *N*_weak_ and $\bar{N}$_all_ = $\bar{N}$_strong_ + $\bar{N}$_weak_). The percentage “correct (strong)” corresponds to *N*_strong_ / (*N*_strong_ + $\bar{N}$_strong_), the fraction of all strong FLYA assignments that have a reference assignment and are correct. The percentage “completeness (strong)” corresponds to *N*_strong_ / (*N*_all_ + $\bar{N}$_all_), the fraction of all FLYA assignments that have a reference assignment and are both strong and correct. The percentage “strong” corresponds to the fraction of all FLYA assignments that are classified as strong. Note that this quantity can be calculated without knowledge of the manual reference assignment. The percentage “correct (weak)” corresponds to *N*_weak_ / (*N*_weak_ + $\bar{N}$_weak_), the fraction of all weak FLYA assignments that have a reference assignment and are correct. The percentage “correct (all)” corresponds to *N*_all_ / (*N*_all_ + $\bar{N}$_all_), the fraction of all FLYA assignments that have a reference assignment and are correct. The values in the table are the median over all proteins for which the given set of spectra is available.

‡Backbone amide groups are considered correctly assigned only if both constituting atoms (H and N) are assigned correctly.

### Complete backbone and side-chain chemical shift assignment

We selected dataset 17 as the minimal spectra set for the complete (backbone and side-chain) assignment of a protein. It comprises the 3D ^15^N- and ^13^C-edited [^1^H,^1^H]-NOESY experiments and the 2D [^1^H,^15^N]-HSQC spectrum (Fig. 1 and Table 1). Assignment results for this dataset are depicted in Fig. 1 and fig. S4. Above the encircled dataset ID (17), the number of proteins in the set is given (84), while the three numbers below report the median percentages of correct assignments obtained if ARTINA is used either without 3D structure input (87.40%, black), with 3D structure input solely for the generation of expected NOESY cross peaks (90.24%, magenta), or when the 3D structure is used also to predict chemical shift distributions for FLYA with UCBShift (90.95%, green). These percentages refer to the strong FLYA assignments. In the second row of the text below each node in the graph, the corresponding numbers are also given for all (strong and weak) FLYA assignments (76.88, 84.77, and 85.86%). This shows that, on average over 84 proteins, most of the chemical shifts can already be assigned using only NOESY as the sole 3D spectra, confirming, on a much larger basis, earlier findings (*20*, *21*). Using the predicted 3D structure, the accuracy of all assignments from the minimal dataset 17 is 5.51 percentage points (pp) lower than for the full set of spectra, dataset 1, used without structure (bottom of Fig. 1 and fig. S4). As expected, use of the 3D structure has greater impact on the assignment accuracy for the NOESY-only minimal dataset 17 (8.98 pp for all assignments) than for the full dataset 1 (1.82 pp).

Starting from the minimal dataset 17, additional spectra can be added in different order. Four sensible ways to expand the number of spectra are shown along the four vertical paths in Fig. 1. In all cases, we first added the 2D [^1^H,^13^C]-HSQC (dataset 16), which requires minimal measurement time and improves the assignment accuracy by 0.46 to 0.82 pp. It is important to note that the number of proteins that are available in each dataset varies, which can influence the assignment accuracy values reported in Fig. 1 and Table 2. To quantify the impact of adding a spectrum for the same set of proteins, Fig. 1 reports, next to the arrows (graph edges), the changes in assignment accuracy between two adjacent datasets computed for the same proteins, i.e., all those for which the spectra of both datasets are available. These changes can (slightly) deviate from the difference between the assignment accuracies for the datasets reported below the colored circles (graph nodes), which have always been calculated for all proteins that match the given spectra set.

From this point on, four strategies can be envisaged (parallel vertical paths from left to right in Fig. 1): (i) classical backbone assignment with HNCA and CBCAcoNH spectra, (ii) simplified backbone assignment with only CBCAcoNH, (iii) backbone assignment only through C^α^ using HNCA and HNcoCA, and (iv) backbone assignment using HNCO and CBCANH instead of CBCAcoNH. In all cases, HCCH-TOCSY and additionally CCH-TOCSY spectra are used as specific side-chain assignment spectra. Last, all paths converge at the full dataset 1, which yields the highest percentage of correct assignments for a given type of usage of the 3D structure.

The results in Fig. 1 indicate that the CBCAcoNH spectrum provides a good balance between information content and measurement time. Expanding dataset 16 with CBCAcoNH [dataset 14 on path (ii)] yields more accurate assignments than expansion with HNCA [dataset 13 on path (i)] or HNCA and HncoCA [dataset 15 on paths (iii) and (iv)]. The CBCAcoNH spectrum alone plus HCCH-TOCSY and CCH-TOCSY for side-chain assignments (dataset 3 with five 3D spectra) delivers better assignment accuracy in structure-based assignment (95.48% correctness for the strong assignments) than the full dataset 1 (9.8 3D spectra on average) in the absence of a structure (94.26%). Using AlphaFold structures is thus equivalent to measuring about five additional 3D spectra. For comparison, using dataset 3 without AlphaFold structures yields 92.77% assignment correctness (Fig. 1). This shows that structures predicted by AlphaFold can reduce the number of NMR spectra required without compromising assignment accuracy. We therefore recommend the experiment types included in dataset 3, comprising two 2D spectra—[^1^H,^15^N]-HSQC and [^1^H,^13^C]-HSQC—as well as five 3D spectra—^15^N-edited [^1^H,^1^H]-NOESY, ^13^C-edited [^1^H,^1^H]-NOESY, CBCAcoNH, HCCH-TOCSY, and CCH-TOCSY—as the optimal spectra set for structure-based chemical shift assignment with ARTINA. While spectrum types included in datasets 2, 4, and 5 on the other paths can be used in principle, they yield slightly lower assignment accuracies and require, in the case of datasets 2 and 5, more spectra than the recommended dataset 3.

Enlarging dataset 3 with further spectra yields, despite substantially longer NMR measurement times, only a marginal improvement of the accuracy of structure-based assignments, i.e., from 95.48% for the recommended dataset 3 to 95.87% for all proteins in the all-spectra dataset 1, or by 0.82 pp for the 41 proteins that are available in datasets 3 and 1. The different spectra in dataset 3 contribute to the structure-based assignment accuracy as follows (if added in this order; see edges in Fig. 1): [^1^H,^13^C]-HSQC, 0.46 pp; CBCAcoNH, 1.48 pp; HCCH-TOCSY, 1.46 pp; CCH-TOCSY, 0.70 pp. This indicates that the next best smaller spectra set is dataset 7, which does not require the CCH-TOCSY spectrum.

The structure, and how it is used, has considerable impact on the assignment. For the recommended dataset 3, the accuracy without structure is 92.77%, which rises to 94.94% if the structure is used only to generate expected NOESY cross peaks, and 95.48% if the structure is also used for shift prediction with UCBShift. Similar observations can be made for other datasets. Since both the use of AlphaFold and UCBShift are “for free,” i.e., do not require additional experimental input data, it is recommended to use predicted structures and shifts for automated chemical shift assignment with ARTINA. Obviously, the help given by the structure depends on its accuracy, i.e., how close it is to the actual structure of the protein in solution. We will address this question below. Using the AlphaFold structure yields equally correct assignments as if the deposited PDB structure is used as input (fig. S6).

The assignment accuracy for all atom types in the 20 standard amino acids is reported in tables S2 and S3 and visualized in fig. S7. The accuracy is highest for the backbone, C^β^/H^β^, and the side chains of the methyl-containing residues Ala, Ile, Val, the Pro ring (except H^γ^), and the side-chain NH_2_ group of Asn. It is lowest for aromatic resonances of His and Phe (C^ɛ^/H^ɛ^ and C^ζ^/H^ζ^), the methyl group of Met, and N^ɛ^/H^ɛ^ of Arg.

The results reported in Fig. 1 and Table 2 also allow the re-evaluation of the quality of the assignment classification as strong or weak by the FLYA algorithm used in ARTINA (*7*). Taking the recommended dataset 3 as an example in Table 2, typically less than 1 in 20 of the strong assignments are erroneous, whereas only up to half of the weak assignments are correct. The strong/weak classification therefore achieves its aim of labeling (almost) as many as possible of the correct assignments as strong. As both classes of assignments are returned by ARTINA, the user may either choose to use only the strong assignments, which we recommend when the accuracy of individual assignments is important (e.g., for deposition in the BMRB, or atom-specific studies of dynamics or interactions), or all (strong and weak) assignments, if it is important to use as many assignments as possible. The latter is most relevant for NMR structure calculations, where it has been shown that for the automated assignment of NOE-based distance restraints with CYANA (*22*, *23*), a missing assignment is almost as bad as an erroneous one (*24*, *25*), and it is therefore better to use a tentative assignment than no assignment at all.

### Assignment of backbone amide groups

Many NMR studies do not require the complete assignment of a protein. Instead, it often suffices to assign the backbone amide groups (HN assignment). We have investigated two approaches to this scenario: HN assignment based on (i) standard triple-resonance backbone assignment spectra (Fig. 2A) and (ii) NOESY-based HN assignment, optionally complemented with backbone assignment spectra (Fig. 2B). Here, we re-evaluate the ARTINA assignment calculations that were performed with the spectra datasets of Table 1 with respect to the accuracy of the backbone amide group assignments. The assignment of an HN group is considered correct only if both ^1^H and ^15^N chemical shifts are assigned correctly within their tolerance of 0.03 and 0.4 ppm, respectively.

If exclusively through-bond backbone assignment spectra are used (Fig. 2A), then the AlphaFold structure can only be exploited to predict chemical shifts with UCBShift. Nevertheless, use of the structure has a strong positive effect on the HN assignment accuracy, improving it by more than 30 pp for some spectra sets. We chose dataset 23 as the minimal spectra set for HN assignment. Dataset 23 comprises the HNCA and [^1^H,^15^N]-HSQC spectra. It is, however, not sufficient to reliably assign the HN groups, as shown by low-average assignment accuracies of 39.13 and 75.00% when used without and with structure input. To achieve high assignment accuracy, it is necessary to expand the set of spectra by CBCAcoNH and CBCANH (dataset 19), which yields 96.39% correct HN assignments if the structure is used. This value is still more than 2 pp lower than the corresponding accuracies obtained with the full dataset 1 without (98.77%) or with (98.98%) structure.

Alternatively, NOESY spectra can be used for structure-based HN assignment (Fig. 2B). In this case, the minimal dataset 25 consists of the 3D ^15^N-edited NOESY and [^1^H,^15^N]-HSQC spectra, which can be measured without ^13^C-labeling of the protein. Dataset 25 yields already 93.67% correct HN assignments if the AlphaFold structure is used. Complementing by CBCAcoNH rises the accuracy to 97.22%. Alternatively, adding 3D ^13^C-edited [^1^H,^1^H]-NOESY (dataset 17) shows that 98.00% accuracy can be achieved purely with 3D NOESY spectra, which is within 1 pp of the highest accuracy obtained by using the full dataset 1 of all available spectra. Using NOESY spectra is thus a viable alternative to dedicated backbone assignment spectra for structure-based HN assignment. Using a 3D structure from AlphaFold increases the HN assignment accuracy by 3.1 pp from 94.89 to 98.00% for the NOESY-only dataset 17. We note that even in the absence of a structure, the NOESY-only approach of dataset 17 yields a slightly higher HN assignment accuracy (94.89%) than the classical triple-resonance approach of dataset 19 (94.57%).

On the basis of the results in Fig. 2 (A and B) and Table 2, we thus recommend the NOESY-only dataset 17 for backbone amide group assignment with ARTINA. Using exclusively through-bond spectra, the recommended (but slightly less accurate) alternative is dataset 19 comprising the three triple-resonance spectra HNCA, CBCAcoNH, and CBCANH.

### Assignment accuracy estimate

**Fig. 3.**
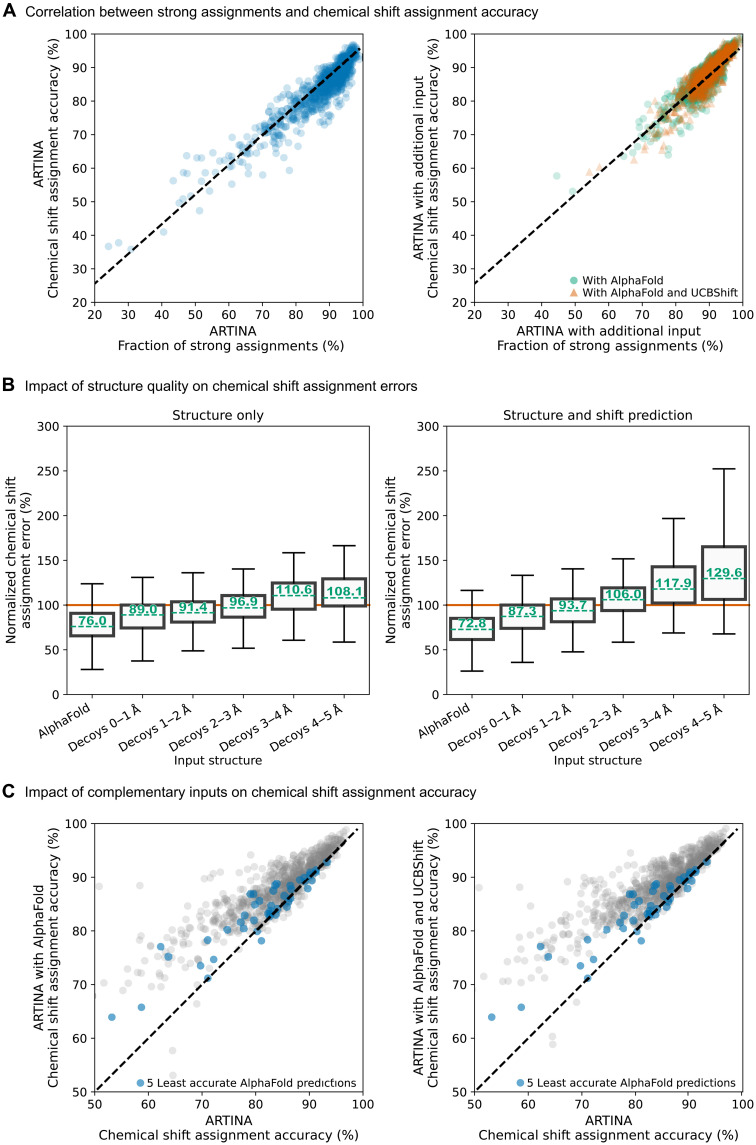
Factors affecting chemical shift assignment accuracy in the proposed integrative approach. (**A**) Correlation between the percentage of strong assignments and chemical shift assignment accuracy, comparing ARTINA-only calculations (left) to those incorporating complementary inputs (right). The strong assignment fraction serves as a reliable indicator of overall assignment accuracy across various experimental settings. The Pearson correlation coefficient [calculated jointly for all input types in (A)] is 0.9046, with a regression model formula of 0.89*x* + 7.88. (**B**) Analysis of the impact of structure quality on chemical shift assignment errors, represented by box plots of normalized assignment errors. These errors are calculated as the ratio between the assignment error in a given experimental configuration and the ARTINA assignment error without any complementary input. For instance, using an AlphaFold structure along with UCBShift predictions as additional input results in a 27.23% reduction in error compared to using ARTINA with spectra and sequence data alone. The orange line at 100% denotes the performance of ARTINA without any complementary input. (**C**) Improvement in chemical shift accuracy due to the inclusion of complementary inputs, such as AlphaFold and UCBShift predictions. Blue dots are for the five proteins with least accurate AlphaFold structure predictions.

The evaluation of assignment results in the two preceding sections relied on comparison with the previously and independently determined reference assignments that are available from the BMRB. In a typical application of ARTINA for the chemical shift assignment of a protein that has not been assigned previously, this would obviously not be possible. Instead, a useful estimate of the assignment accuracy obtained from the experimental data without recourse to a reference assignment can be derived from the percentage of strong assignments reported by FLYA. Figure 3A shows a clear and universal correlation between the percentage of strong assignments and the assignment accuracy that holds over more than 5000 assignment calculations with 25 datasets for 89 proteins, with and without using a structure. On the basis of this correlation, an estimate *A* of the accuracy of the assignments can be obtained from the percentage *S* of strong assignments according to the linear relationship *A* = 0.89 *S* + 7.88%. A similar estimate, *A*^strong^ = 0.45 *S* + 51.49%, applies for the accuracy of the strong assignments.

### Impact of structure accuracy on the assignment accuracy

Ordinarily, the precision of AlphaFold allows for protein structure predictions that rival those derived experimentally (*16*), as is the case for all proteins in our test dataset (table S1). Nevertheless, in principle, serious deviations can occur. We therefore investigated how the accuracy of input structures influences structure-based chemical shift assignment with ARTINA. For this purpose, we artificially generated many decoy structures with backbone RMSD in the range of 0 to 5 Å by applying 3DRobot (*18*) to the PDB reference structures. Comparably to AlphaFold structures, these decoys remain generally well packed, contain parts or all of the native secondary structure elements, and are devoid of obvious errors such as severe steric clashes.

We used decoys to predict chemical shifts with UCBShift. Subsequently, ARTINA assignment calculations were conducted with these decoy structures and, optionally, additional UCBShift predictions (Fig. 3B). For comparability, relative assignment error rates, normalized by the error rate of the corresponding assignment obtained by ARTINA without input structure, are shown in Fig. 3B. For instance, if, for a given set of spectra and protein, the assignment accuracy is 90% using the given input structure and 75% without input structure, the relative error rate is (100 − 90)/(100 − 75) = 40%. Relative error rates below 100% indicate that the use of the structure improves the assignment.

The use of the AlphaFold structure substantially improves the assignment accuracy, with median relative error rates of 76.02%. This improvement is also observed with decoy structures that have backbone RMSD under 3 Å to the reference PDB structure (median relative error rates of 88.99 to 96.88%), although the advantage decreases with decreasing accuracy of the input structures. Decoy structures with an RMSD between 0 and 2 Å exhibit the best performance, having median relative error rates of 88.99 to 91.43%. In contrast, structures with an RMSD within the 2- to 3-Å range provide minimal help (96.88%), and those with larger deviations tend to detrimentally affect the assignment process.

Figure 3B further illustrates that incorporating chemical shift prediction with UCBShift as an additional step makes the workflow more susceptible to the accuracy of the input structure, which is the consequence of error accumulation. Compared to ARTINA runs without any additional input, the integration of shift predictions proves advantageous for AlphaFold structures and the decoys with backbone RMSD under 2 Å. However, a close examination of the plots in Fig. 3B reveals that even though decoys with RMSD 1 to 2 Å yield a higher-average assignment accuracy than ARTINA without a structure, it is more effective to use the structure model only for expected NOESY peak generation, skipping the UCBShift prediction step (normalized error 91.43%, compared to 93.67%).

An additional finding is that AlphaFold structures offer better support for ARTINA assignments than decoys. AlphaFold structures outperform the finest decoys (Fig. 3B). This may be due to better side-chain packing in the AlphaFold structures, which is beneficial for the full protein assignment but does not improve the backbone RMSD presented on the plot’s horizontal axis. To further understand the assignment performance with AlphaFold structures, we examined the chemical shift accuracy of all proteins, both with and without complementary inputs, as presented in Fig. 3C. They clearly demonstrate a robust positive impact when AlphaFold or AlphaFold combined with corresponding UCBShift predictions are used, as reflected by data points above the diagonal. This improvement in assignment quality is observed even when considering only the bottom 5% of the AlphaFold predictions that deviate most from the corresponding reference structure.

### Assignment of large proteins

**Fig. 4.**
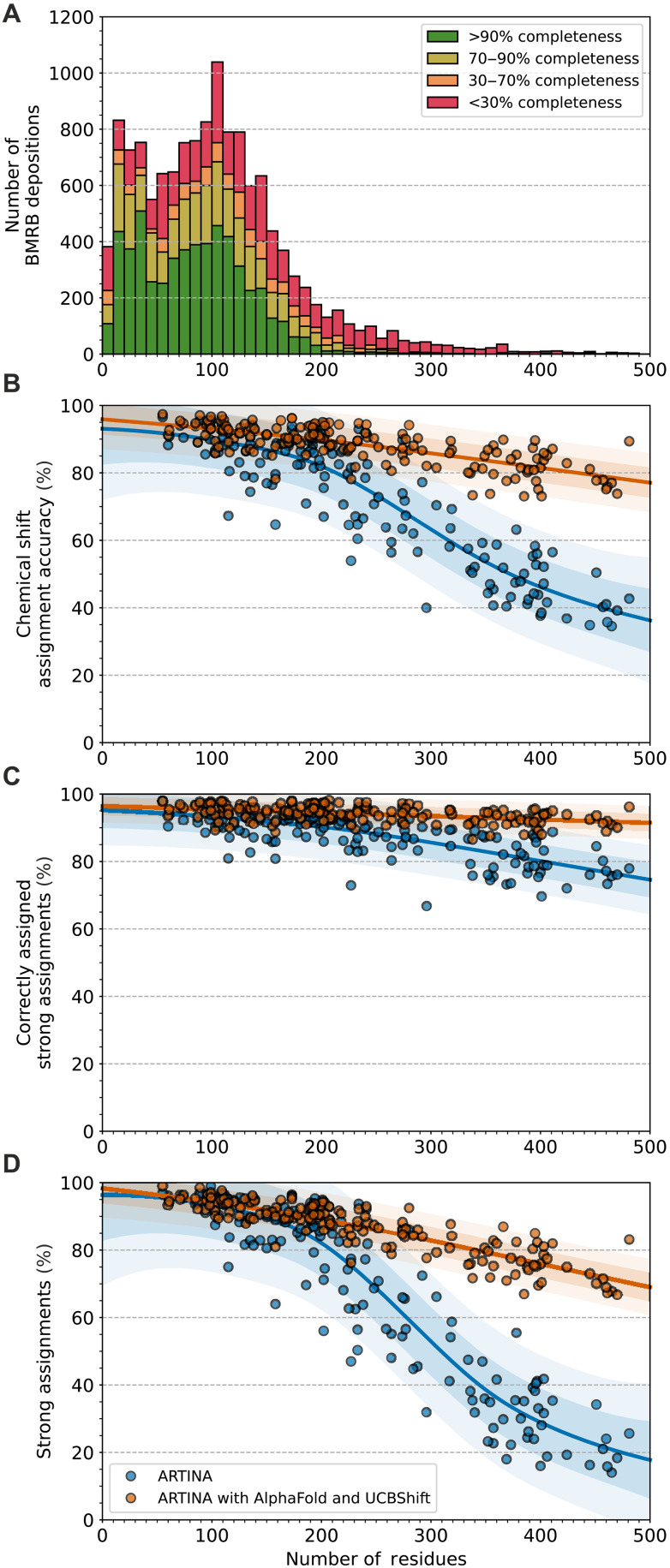
Impact of protein size on the accuracy of automated chemical shift assignment. (**A**) Completeness of protein backbone amide ^1^H and aliphatic ^1^H chemical shift assignments as a function of protein size in all BMRB depositions available on 10 May 2023. (**B**) Accuracy of full (backbone and side-chain) chemical shift assignment. (**C**) Accuracy of strong backbone and side-chain shift assignment. (**D**) Fraction of chemical shifts assignments classified as strong by FLYA. Each data point in (B) to (D) represents the result of an automated chemical shift assignment of a protein with the recommended dataset (dataset 3), both with (orange) or without (blue) complementary AlphaFold and UCBShift predictions. Regression lines and ± σ and ± 2σ confidence intervals (semitransparent shaded areas) were obtained by Gaussian process regression with a Matérn kernel.

Assigning NMR spectra of large proteins with more than 200 residues presents a fundamental challenge in the field of NMR spectroscopy, due to several factors affecting both measurement process (line broadening, lower sensitivity, and larger conformational heterogeneity) and data interpretation (signal overlap and shift assignment ambiguity). The cumulative effect of these factors is illustrated in Fig. 4A, which shows an exponential decay in the number of assigned proteins deposited in BMRB, beginning from 150 residues, despite their biological significance and substantial interest in NMR studies (*26*, *27*), and corroborating earlier observations (*28*). The introduction of 1.2-GHz NMR spectrometers has expanded the limits of NMR spectroscopy in terms of resolution (*29*), paving the way for the further development of computational methods. This progress serves as the underlying motivation for evaluating the performance of our previous method, ARTINA, and our integrative approach in context of potential large-protein assignments.

For this study, we prepared data as follows. First, we selected 41 proteins from the ARTINA benchmark, for which ^13^C/^15^N-edited [^1^H,^1^H]-NOESY, CBCAcoNH, HCCH-TOCSY, and CCH-TOCSY experiments are available (the recommended spectra subset). Second, we randomly selected 155 protein subsets (typically one to four proteins in each subset), ensuring that each subset contains up to 500 residues in total. For each protein subset, we concatenated corresponding AlphaFold predictions using 10 glycine linkers (fig. S3). This step necessitated structure regularization with CYANA (*30*), during which protein individual structures were kept fixed and only linker coordinates were refined to avoid structural clashes in the concatenated model. Furthermore, we aligned and combined chemical shift lists (BMRB depositions and UCBShift predictions) and experimental peak lists accordingly to the concatenated AlphaFold structures. It is important to note that in this in silico experiment, we used experimental cross peak coordinates extracted automatically from NMR spectra (each spectrum representing a separate domain in the concatenated model), but the automated chemical shift assignment was performed on the concatenated peak lists. Although this constitutes a further simplification compared to the experimental setting, where all domains are recorded simultaneously in a single spectrum, the experiment allows to assess the impact of the system size on the accuracy of the chemical shift assignment.

With such multidomain protein datasets, we carried out full chemical shift assignment (backbone and side-chain) using ARTINA either without any complementary inputs (Fig. 4, B to D, blue) or with both AlphaFold and UCBShift predictions (Fig. 4, B to D, orange). As illustrated in Fig. 4B, when the ARTINA workflow is executed using only spectra and sequence, its performance begins to decline rapidly once the system size exceeds approximately 200 residues. This decline can be attributed to the combinatorial complexity of the optimization problem being solved and the ambiguity of the cross peak assignments. The overall accuracy of the chemical shift assignment decreases from 89.76 to 36.27% as the system size expands from 100 to 500 residues. In contrast, the integrative approach alleviates this issue, achieving assignment accuracies 93.06 and 77.09% for 100 and 500 residue proteins, respectively, and maintaining an assignment accuracy above 80% for systems up to 432 residues.

Our previous experiments have confirmed that strong assignments can be considered reliable (Table 2). This statement was supported by large-scale experiment with more than 5000 assignment calculations and 89 proteins of size up to 175 residues. Upon examining the characteristics of ARTINA and FLYA with larger systems, it becomes evident that the quality of strong assignments declines less with increasing protein size than the overall assignment accuracy (Fig. 4C), reaching 74.58% for assignments where ARTINA was used without any complementary inputs (compared to 36.27% for all shifts) and 91.55% for ARTINA with AlphaFold and UCBShift predictions (77.09%). This observation leads to the conclusion that when ARTINA is used with complementary inputs, its strong assignments remain reliable, even for large systems. Nevertheless, the fraction of observed strong assignments varies depending on the used experimental setting (Fig. 4D).

## DISCUSSION

In this work, we propose an integrative approach to deep learning–based automated chemical shift assignment of proteins, which combines our previous method ARTINA with AlphaFold and UCBShift, outperforming previously reported results in terms of accuracy, efficiency, and robustness of the automated chemical shift assignment. In comprehensive studies with more than 5000 automated assignment calculations on 89 proteins, the impact of quantity and quality of the input data for assignment has been investigated, yielding practical recommendations for rapid automated analysis of NMR spectra. In addition, we pointed out the fundamental advantage of the integrative approach for studies of large proteins by NMR. Last, the proposed integrative approach paves the way to backbone group assignment using only ^15^N-labeled samples, which provides an alternative to costly research with ^13^C labeling. A quantitative summary of the above statements is presented in Table 3.

**Table 3.** Quantitative summary of key chemical shift assignment experiments.

Assignment task	Spectra used in the experiment		Dataset ID	Accuracy* (%)	
	2D	3D		Standard approach	Integrative approach
Full (backbone and sidechains)	[^1^H,^15^N]-HSQC [^1^H,^13^C]-HSQC	^15^N-edited [^1^H,^1^H]-NOESY, ^13^C-edited [^1^H,^1^H]-NOESY, CBCAcoNH, HCCH-TOCSY, and CCH-TOCSY	3	92.77 (89.01)	95.45 (92.59)
Backbone HN groups (^13^C and ^15^N labeling)	[^1^H,^15^N]-HSQC	^15^N-edited [^1^H,^1^H]-NOESY and ^13^C-edited [^1^H,^1^H]-NOESY	17	94.89 (84.13)	98.00 (94.40)
Backbone HN groups (^15^N labeling only)	[^1^H,^15^N]-HSQC	^15^N-edited [^1^H,^1^H]-NOESY	25	87.50 (26.96)	93.67 (83.59)
Full (backbone and sidechains) proteins ~100 residues	[^1^H,^15^N]-HSQC [^1^H,^13^C]-HSQC	^15^N-edited [^1^H,^1^H]-NOESY, ^13^C-edited [^1^H,^1^H]-NOESY, CBCAcoNH, HCCH-TOCSY, and CCH-TOCSY	3	93.34 (89.76)	95.50 (93.06)
Full (backbone and sidechains) proteins ~500 residues				74.58 (36.27)	91.55 (77.09)

*Accuracy refers to the median of the accuracy of the assignments obtained for the proteins in our study. The first number is for strong assignments, and the number in parenthesis is for all assignments. See footnotes of Table 2 for details.

The proposed integrative approach is more efficient than ARTINA without structure input in extracting information from NMR spectra due to reduced ambiguity of the chemical shift assignment. On average, five 3D spectra (^15^N-edited [^1^H,^1^H]-NOESY, ^13^C-edited [^1^H,^1^H]-NOESY, CBCAcoNH, HCCH-TOCSY, and CCH-TOCSY) are sufficient to assign correctly 95.45% of the chemical shifts, which outperforms previously reported results for ARTINA using all available spectra but no AlphaFold and UCBShift predictions (91.37%). An even higher median accuracy of 94.40% can be achieved for the backbone amide groups using just two 3D spectra, ^15^N-edited [^1^H,^1^H]-NOESY and ^13^C-edited [^1^H,^1^H]-NOESY. Backbone amide group assignment works slightly better with the NOESY spectra than with dedicated triple-resonance backbone assignment spectra. Considering that the NOESY spectra provide a wealth of other relevant information, e.g., about the conformation or multiple states of a protein (*19*), whereas the triple-resonance through-bond spectra have little use beyond establishing the assignment, this renders NMR studies more efficient since the spectra can be used simultaneously for assignment and other purposes.

Regarding the studies with only ^15^N-labeled samples, the original ARTINA approach could not assign backbone amine chemical shifts to a practically relevant extent (26.96% correct among all assignments). In contrast, the integrative approach yielded substantially more correct assignments (83.59% of all assignments). Being able to assign backbone amide groups with a minimal number of spectra and without ^13^C-labeling facilitates efficient studies of protein interactions and dynamics.

ARTINA proposes an assignment for every atom that has been used to assign at least one peak in one of the spectra. These assignments are then classified as “weak” (tentative) or “strong” (reliable), and it has been shown earlier (and is confirmed by Table 2) that there are about 10 times fewer errors among the strong assignments than among the weak ones. Nevertheless, the error rate in not zero even for strong assignments (as, presumably, there may also be a small number of errors in deposited manually determined assignments). In general, these errors have little impact on a subsequent structure calculation but may be a problem for other NMR studies that rely on the correctness of individual assignments. In that case, manual verification may be applied to detect and fix potentially unsafe assignments, a task that is greatly simplified by the assigned peak lists and chemical shift lists provided by ARTINA.

Studies of intrinsically disordered regions and proteins are an important application domain for NMR spectroscopy that poses challenges for both manual and automated analysis. The ARTINA benchmark contains only folded proteins with mainly well-defined structure. Nevertheless, also these proteins comprise not well-structured and unstructured regions, e.g., near chain ends or in loops. To obtain an estimate for the performance of our method regarding intrinsically disordered regions, we calculated the chemical shift assignment accuracy for the subsets of atoms that are either within or outside well-structured regions, as defined by the CYRANGE algorithm (*31*) and reported earlier (*11*). Results are given in tables S4 and S5. As expected, our method performs better within well-structured regions (median accuracy 92.71% for the full dataset and 92.62% for the recommended dataset) than outside (median accuracy 86.84% for the full dataset, 84.37% for the recommended dataset). Using AlphaFold structures and UCBShift predictions as input increases the overall chemical shift assignment accuracy in both types of regions, e.g., by 4.94 pp within well-structured regions versus 4.38 pp outside well-structured regions for the recommended spectra dataset.

The integrative approach is expected to be particularly advantageous in NMR studies of 200- to 500-residue proteins, which are of high interest in structural biology but severely underrepresented in the BMRB repository. Such proteins pose challenges for both NMR (due to signal overlap, sensitivity and relaxation) and cryo–electron microscopy (contrast issues), resulting in x-ray crystallography as the method of choice. Although unavoidably larger protein size has a negative effect on the integrative approach, the performance of the integrative approach decreases much less (from 93.06% correct assignments for proteins in the 100-residue size range to 77.09% for the simulated proteins in the 500-residue range; Table 3) than for ARTINA without any complementary input (from 89.76 to 36.27%). Having said this, it must be kept in mind that the analysis presented here assesses mainly the effects of increased peak overlap and the larger number of atoms to assign but neglects the increase of relaxation with protein size, which broadens cross peaks and leads to a concomitant further increase of peak overlap. For instance, doubling the protein size from 200 to 400 amino acid residues approximately doubles resonance linewidths. However, it can be expected that a combination of measurements at ultrahigh field of 1.2 GHz, partial deuteration (*32*), as well as further method developments such as the combined ^13^C,^15^N-edited [^1^H,^1^H]-NOESY experiment (*19*) will alleviate this overlap issue. Better resolution of the input spectra can also result from advanced experimental and data processing methods, for example, by homonuclear C^α^-C^β^ decoupling using software deconvolution or a decoupling sequence, which can markedly improve resolution in HNCA spectra (*33*), or by nonuniform sampling techniques (*34*).

In summary, in our previous study (*11*) the main gain in efficiency for protein chemical shift assignment was the machine learning–based, complete automation of the entire process, starting from the uninterpreted spectra, which leaves the NMR measurements as the main time-limiting step. The integrative approach presented in this work constitutes an improvement regarding NMR measurement time, sample preparation, and protein size. Using the small sets of spectra identified in this study, the NMR measurements, and thus the effort and cost for the NMR assignment of a protein can be reduced several-fold, proteins may be labeled only with ^15^N for backbone HN assignment, and larger proteins can likely be assigned, which enlarges the protein family easily accessible by NMR to many biologically and pharmaceutically interesting systems.

The integrative approach has also potential for studies of other types of systems, such as protein complexes, intrinsically disordered proteins, membrane proteins, as well as in-cell and solid-state NMR, for which machine learning methods are equally promising than for the monomeric globular proteins in solution studied with ARTINA so far. Such applications are currently hampered by limited availability of suitable spectra datasets, which we expect to overcome by spectrum simulation for the generation of training data and by facilitating NMR primary data deposition in public databases for the accumulation of experimental test data.

## MATERIALS AND METHODS

The original ARTINA algorithm (*11*) uses as input exclusively a set of multidimensional NMR spectra and the amino acid sequence of the protein. We extended this approach to handle additional types of input data, including 3D protein structures, chemical shifts values or distributions, and peak lists prepared by other methods (not used in this study). The chemical shift information may comprise already known assignments for a subset of atoms (for instance, from manual spectra analysis; not used in this study) and/or statistical information on the distribution of chemical shifts (used here). All sources of input data are optional, with the prerequisite that at least one input spectrum or peak list must be available.

### Integrative approach for structure-based chemical shift assignment

**Fig. 5.**
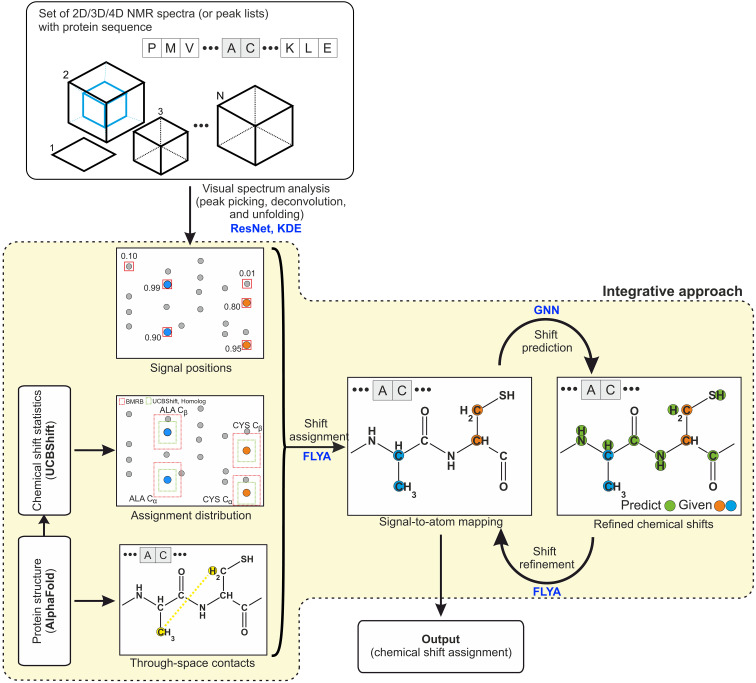
Integrative approach to automated chemical shift assignment. GNN, graph neural network; KDE, kernel density estimation; ResNet, deep residual neural network.

The integrative approach (Fig. 5) uses three complementary sources of information: NMR spectra, 3D structures, and chemical shift distributions. The latter two types of information are derived from predictions that require only the amino acid sequence as input and therefore not any additional measurements. The aim of the integrative approach is to derive more accurate assignments than the original, purely spectra-based ARTINA workflow with a lower demand for experimental NMR data and thus to cut down the required NMR measurements.

NMR spectra are analyzed with a deep residual neural network and, if necessary, unfolded automatically yielding signal positions (Fig. 5). Each detected cross peak has assigned a classifier response in the range [0, 1], which indicates the probability that the signal is a true peak rather than an artifact. Because the protein sequence is known, one can calculate the expected number of cross peaks in each spectrum. This number is used to select cross peaks with highest classifier response and form an input for FLYA chemical shift assignment. In case the number of expected peaks cannot be assessed (i.e., in case of NOESY experiments), a fixed threshold value of 0.1 is used to select the signals. It has been shown to be beneficial to select cross peaks generously and filter out the artifacts at later stages of the analysis because the FLYA automated assignment algorithm can cope better with additional artifact peaks than with missing true peaks (*7*).

For the assignment calculations in this paper, input 3D structures are obtained, unless noted otherwise, by sequence-based structure prediction with AlphaFold using as input the sequence of the protein construct for which the NMR spectra had been recorded. To avoid potential bias, only PDB templates that were deposited in the data bank before the publication of the query protein are used for AlphaFold structure prediction. Five structure candidates predicted by AlphaFold were combined into a structure bundle that resembles NMR models.

To investigate the required accuracy of 3D structures for our integrative approach, we also generated artificially disturbed variants of the PDB structures. We used 3DRobot (*18*) to create 100 well-structured decoys for each benchmark protein used in this study. These decoys cover a range of 0 to 15 Å in backbone RMSD relative to the PDB reference, of which those with RMSD 0 to 5 Å were used for our study. To again assemble structure bundles, we identified triplets of decoys having pairwise backbone RMSD below 1 Å.

The 3D structures are used directly to generate expected NOESY peaks (see below), and indirectly, as input for chemical shift predictions with UCBShift (*17*), which are in turn used to reduce the search range for C^α^ C^β^, C′, H^N^, H^α^, and N chemical shift assignments. This facilitates the assignment by decreasing the number of assignment possibilities that have to be considered during combinatorial optimization. In general, the number of assignment possibilities decreases exponentially with the width of the chemical shift distributions (*35*). Note that the protein structure used in the integrative approach may also be obtained by other methods (x-ray crystallography) or originate from a homologous protein.

### Automated chemical shift assignment with FLYA

The previously described FLYA algorithm (*7*) for automated chemical shift assignment uses a combination of evolutionary and local optimization (*6*) to optimally map cross peaks expected on the basis of sequence and structure to cross peaks observed in the experimental spectra. With this general approach, it can determine chemical shift assignments using peak lists from any combination of multidimensional NMR spectra.

3D structures can be used directly by FLYA when generating the cross peaks that are expected in NOESY spectra. An expected NOESY cross peak is generated whenever the corresponding distance is shorter than 6.0 Å in all conformers in the structure bundle. In the absence of an input structure, FLYA applies this criterion to an internally generated bundle of random structures, i.e., structures with correct covalent geometry but random torsion angle values that are only minimized to avoid steric clashes. Consequently, only expected cross peaks that correspond to short-range distances (within a residue or between neighboring residues) will be obtained because the distance between two atoms located far apart in the protein sequence are highly unlikely to be consistently short in all members of the random structure bundle. In contrast, if a narrow bundle of structures is provided to FLYA, then also medium-range and long-range expected NOESY cross peaks will be generated, which corresponds to the situation in the experimental spectra where such peaks are observed. In FLYA, expected peaks are further characterized by an a priori estimate of the probability to actually observe the peak in an experimental spectrum, which is used for selecting peak mappings and scoring assignments during the optimization (*7*). For NOESY cross peaks, this estimated probability is set to 0.9 if the maximal distance *d*_max_ in the structure bundle is shorter than 4 Å, 0.8 if 4 ≤ *d*_max_ < 4.5 Å, 0.7 if 4.5 ≤ *d*_max_ < 5 Å, 0.6 if 5 ≤ *d*_max_ < 5.5 Å, and 0.5 if 5.5 ≤ *d*_max_ < 6 Å. In “through-bond” spectra, the estimated probabilities are set to fixed values, stored in the CYANA library file, for each magnetization transfer path that can give rise to an expected peak.

Input chemical shift predictions replace, if available, the default statistics used by FLYA as a priori information for the chemical shift assignment, which is derived from the distribution of chemical shift values over all known assignments for a given atom in the BMRB data bank (*36*). Chemical shift distributions are modeled in FLYA as normal distributions defined by their mean and SD. Using structure-based chemical shift prediction, one can in many cases obtain chemical shift distributions that are more accurate (i.e., have a mean value closer to the actual chemical shift value) and more precise (i.e., have a smaller SD then the general BMRB distribution) and thus help FLYA in determining reliable assignments. To achieve this, it is not necessary that the chemical shift prediction algorithm provides the correct value with high accuracy.

The FLYA algorithm yields a chemical shift assignment for all atoms involved in expected cross peaks that are mapped to an observed peak. FLYA classifies these assignments as either strong (reliable) or weak (tentative) by running the assignment algorithm multiple times (20 times for all calculations in this paper) with identical input data but different random numbers to initialize the optimization algorithm (*7*). The assignment of a given atom is classified as strong if at least 80% of the chemical shift values found for this atom in the 20 independent runs fall within a range of ω ± Δω, where ω is the optimal consensus chemical shift value and Δω is a tolerance of 0.03 ppm for ^1^H and 0.4 ppm for ^13^C and ^15^N. Assignments of atoms that do not fulfill this criterion are classified as weak. Weak assignments are often still correct but carry an about 10 times higher chance of being wrong than the strong assignments (*7*).

### Chemical shift refinement with graph neural network and FLYA

In the subsequent step we bring to the workflow information about thousands of structures solved in the past by NMR spectroscopy. This is done through a graph neural network (GNN) that has been trained on the BMRB database, as described earlier (*11*). The model accepts as input molecular graph of the protein with information about chemical shifts classified as strong in each node of the graph. By solving a node regression problem, GNN predicts expected values for all missing (i.e., weakly or not assigned in the preceding FLYA run) chemical shifts given the ones strongly assigned by FLYA. In the proposed approach, we make only one shift prediction and refinement step (Fig. 5), which yields the final output of our procedure. When UCBShift is used, its chemical shift predictions are only used in the first FLYA run, whereas GNN predictions are always used only in the second FLYA run.

### Decoy structure generation

We used 3DRobot (*18*) with standard parameters to create, starting from the PDB reference structure, 100 well-structured decoys for each benchmark protein used in this study. These decoys cover a range of 0 to 15 Å in backbone RMSD relative to the PDB reference. The decoy structures, refined at the atomic level through energy optimization, bear similarities with the true folds deposited in the PDB, such as the quantity and placement of secondary structure elements (fig. S1). To assemble protein bundles, we identified triplets of decoys with pairwise backbone RMSD below 1 Å.
