# Supplementary material for: Time-optimized protein NMR assignment with an integrative deep learning approach using AlphaFold and chemical shift prediction
Source: Sci Adv. 2023 Nov 22;9(47):eadi9323. doi: 10.1126/sciadv.adi9323 (PMC10664993; doi:10.1126/sciadv.adi9323)
Supplement: Supplementary file 1 — Figs. S1 to S7 Tables S1 to S5 [file sciadv.adi9323_sm.pdf]

Science Advances
AAAS

Supplementary Materials for

Time-optimized protein NMR assignment with an integrative deep learning approach using AlphaFold and chemical shift prediction

Piotr Klukowski *et al.*

Corresponding author: Piotr Klukowski, piotr.klukowski@phys.chem.ethz.ch; Roland Riek, roland.riek@phys.chem.ethz.ch; Peter Güntert, peter.guentert@phys.chem.ethz.ch

Sci. Adv. **9**, eadi9323 (2023)
DOI: 10.1126/sciadv.adi9323

This PDF file includes:

Figs. S1 to S7
Tables S1 to S5

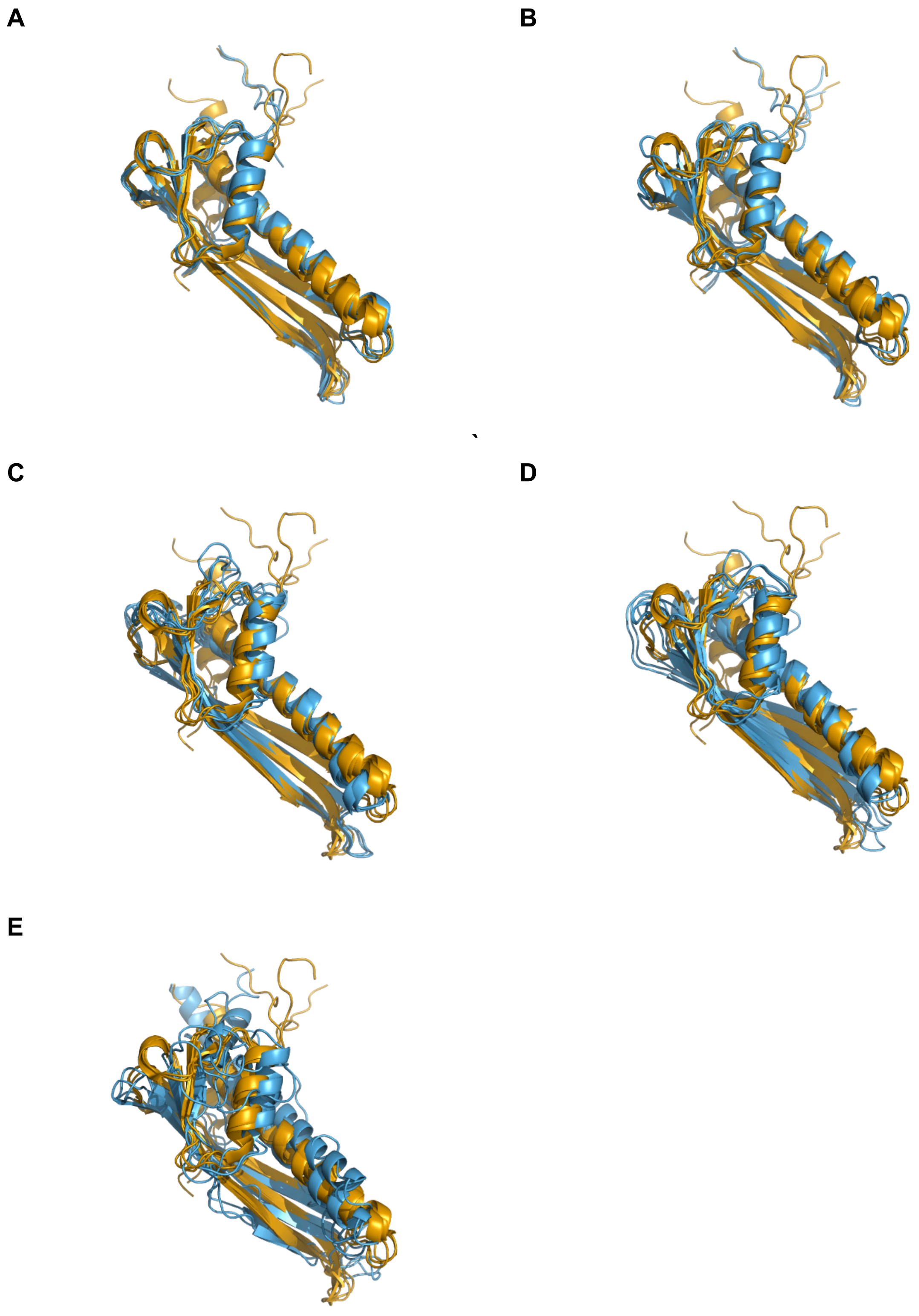

Figure S1. Selected decoys (blue) superimposed on the corresponding experimental PDB structure, 2JXP (orange). (**A**) 0.81 Å backbone RMSD to 2LF2. (**B**) 1.18 Å. (**C**) 2.95 Å. (**D**) 3.06 Å. (**E**) 4.24 Å.

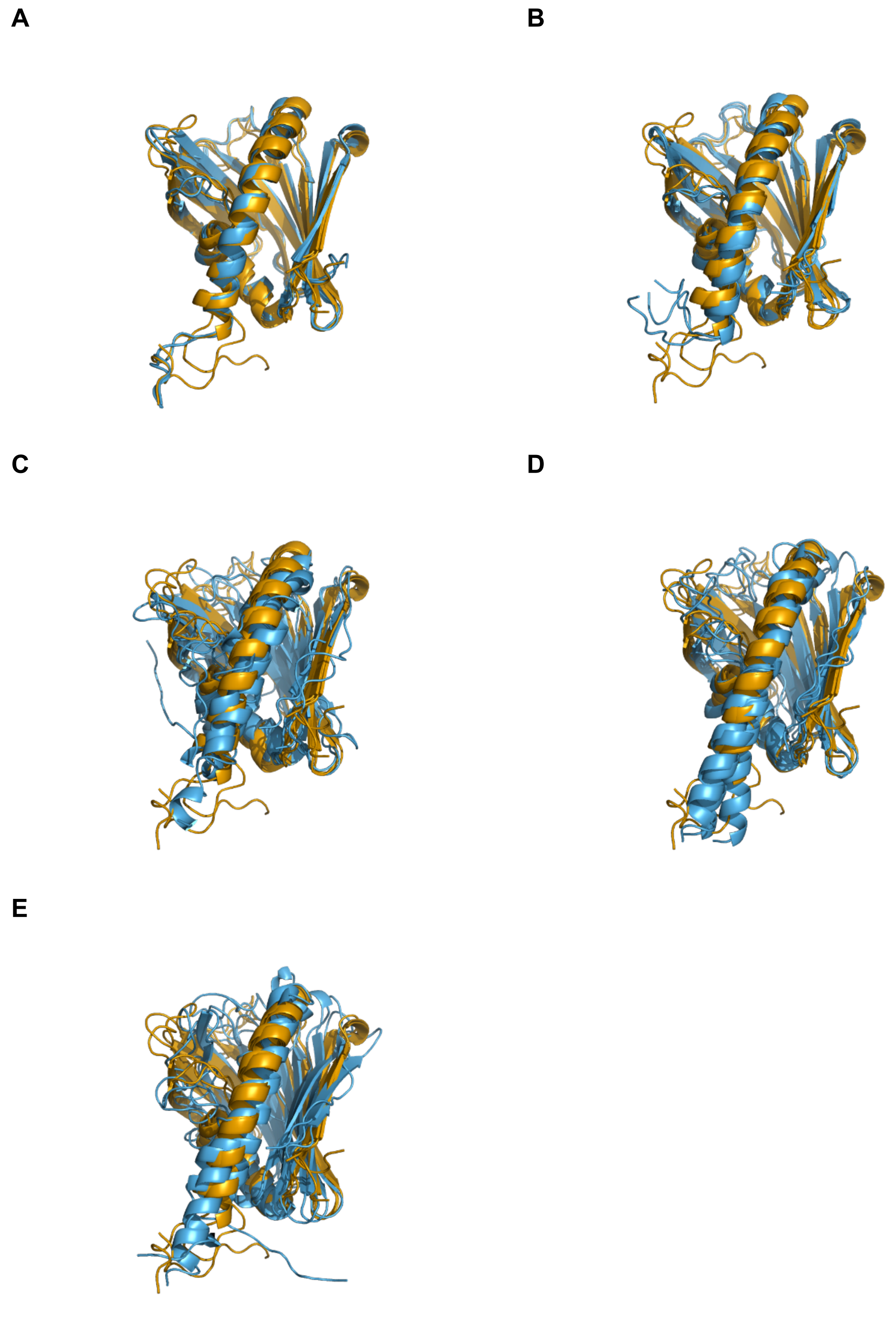

Figure S2. Selected decoys (blue) superimposed on the corresponding experimental PDB structure, 2LF2 (orange). (**A**) 0.82 Å backbone RMSD to 2LF2. (**B**) 1.05 Å. (**C**) 2.07 Å. (**D**) 3.09 Å. (**E**) 4.03 Å.

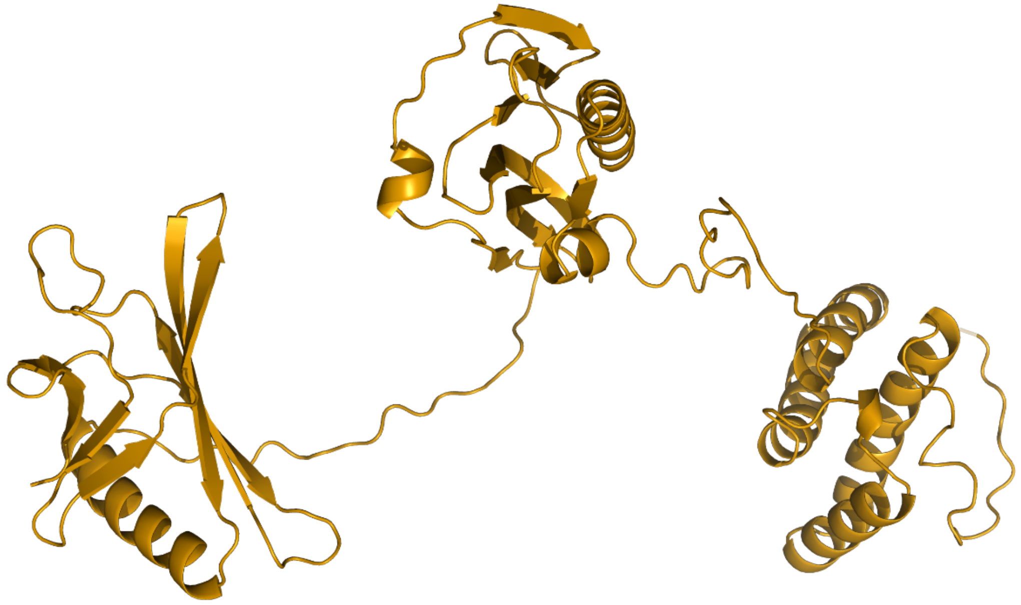

Figure S3. Synthetic example system used for assessing the assignment of large proteins. Three fixed domains correspond to the PDB structures 2KVO, 2KKZ and 2KIW that are connected with 10-glycine linkers. The system has in total 372 residues.

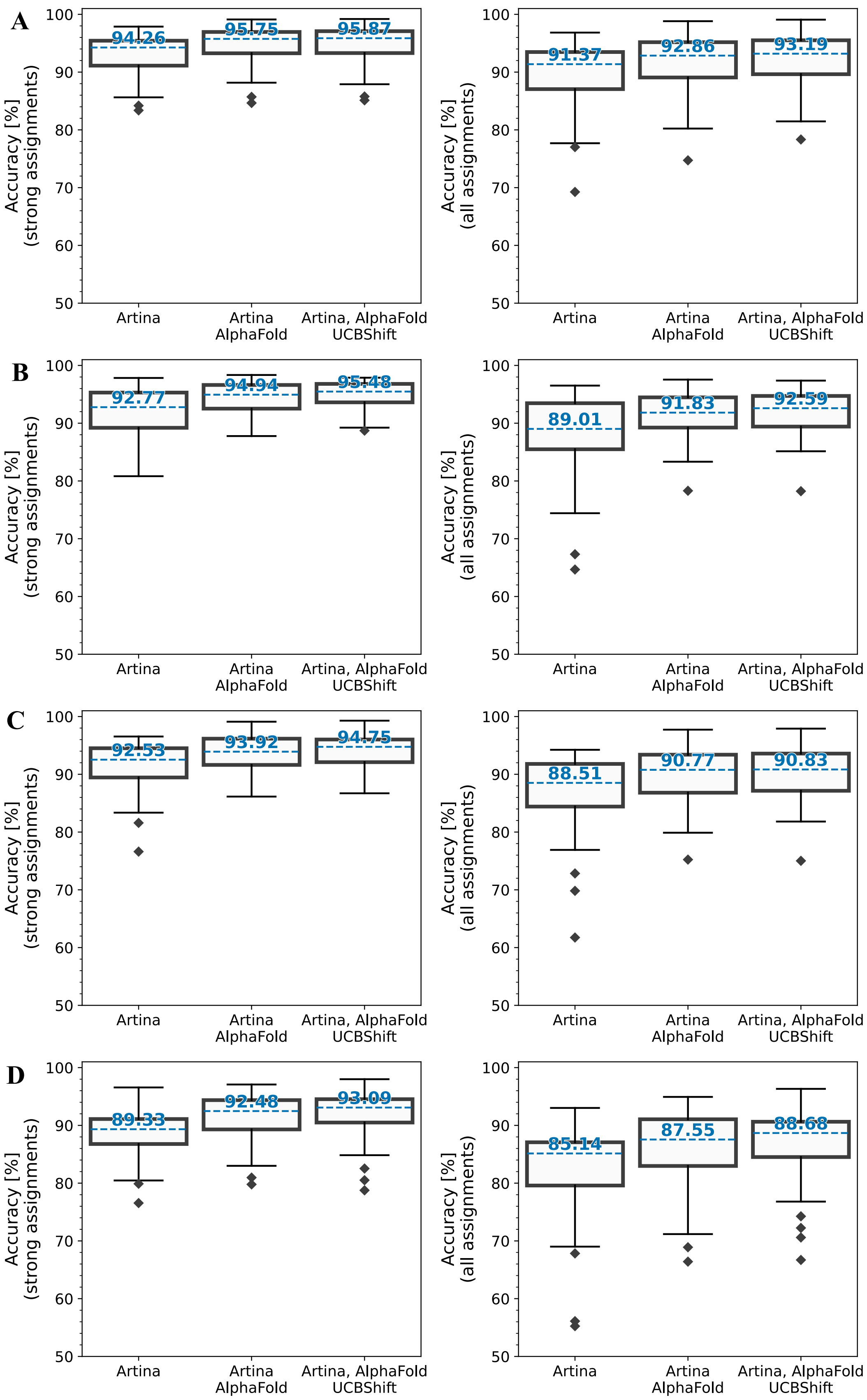
A
B
C
D
Accuracy [%] (strong assignments)
Accuracy [%] (all assignments)
Artina
Artina AlphaFold
Artina, AlphaFold UCBShift
94.26
95.75
95.87
91.37
92.86
93.19
92.77
94.94
95.48
89.01
91.83
92.59
92.53
93.92
94.75
88.51
90.77
90.83
89.33
92.48
93.09
85.14
87.55
88.68

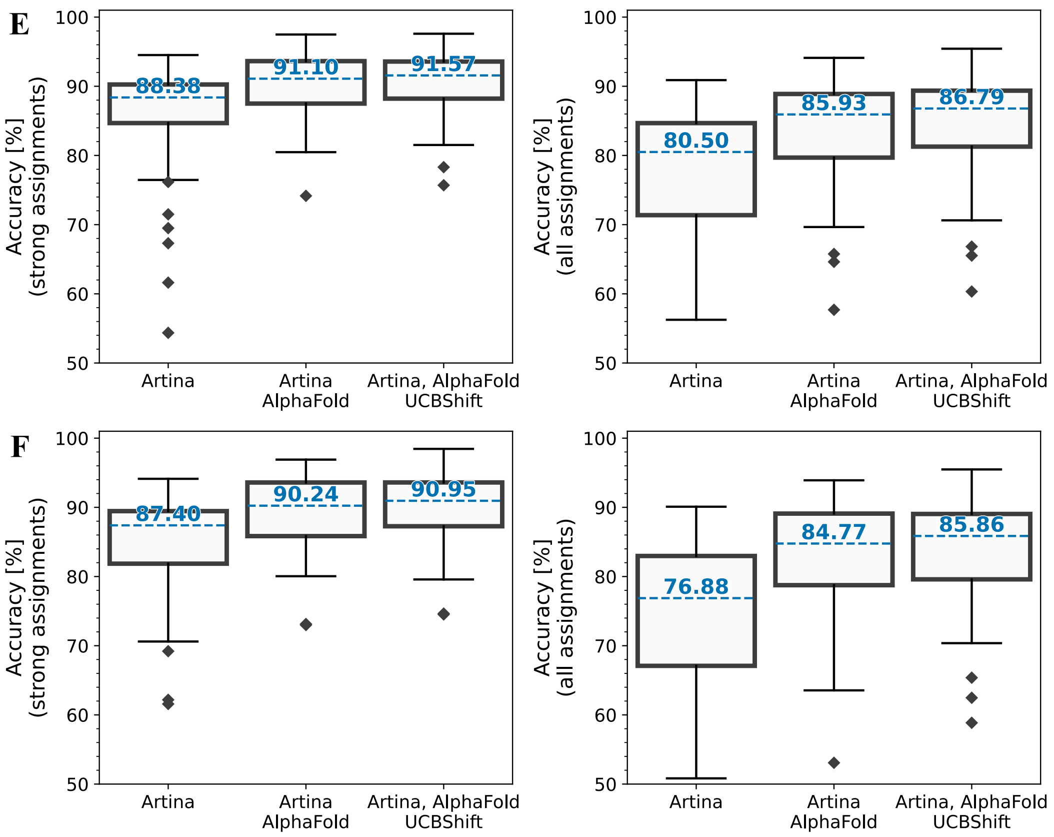

Figure S4. Distribution of chemical shift assignment accuracy for selected spectra subsets. (**A**) Dataset 1. (**B**) Dataset 3. (**C**) Dataset 7. (**D**) Dataset 14. (**E**) Dataset 16. (**F**) Dataset 17. Each box plot illustrates the distribution of chemical shift assignment accuracies, with median values corresponding to those reported under graph nodes in Figure 1. The analysis has been performed for both strong and all assignments for three experimental settings (ARTINA, ARTINA with AlphaFold, ARTINA with AlphaFold and UCBShift).

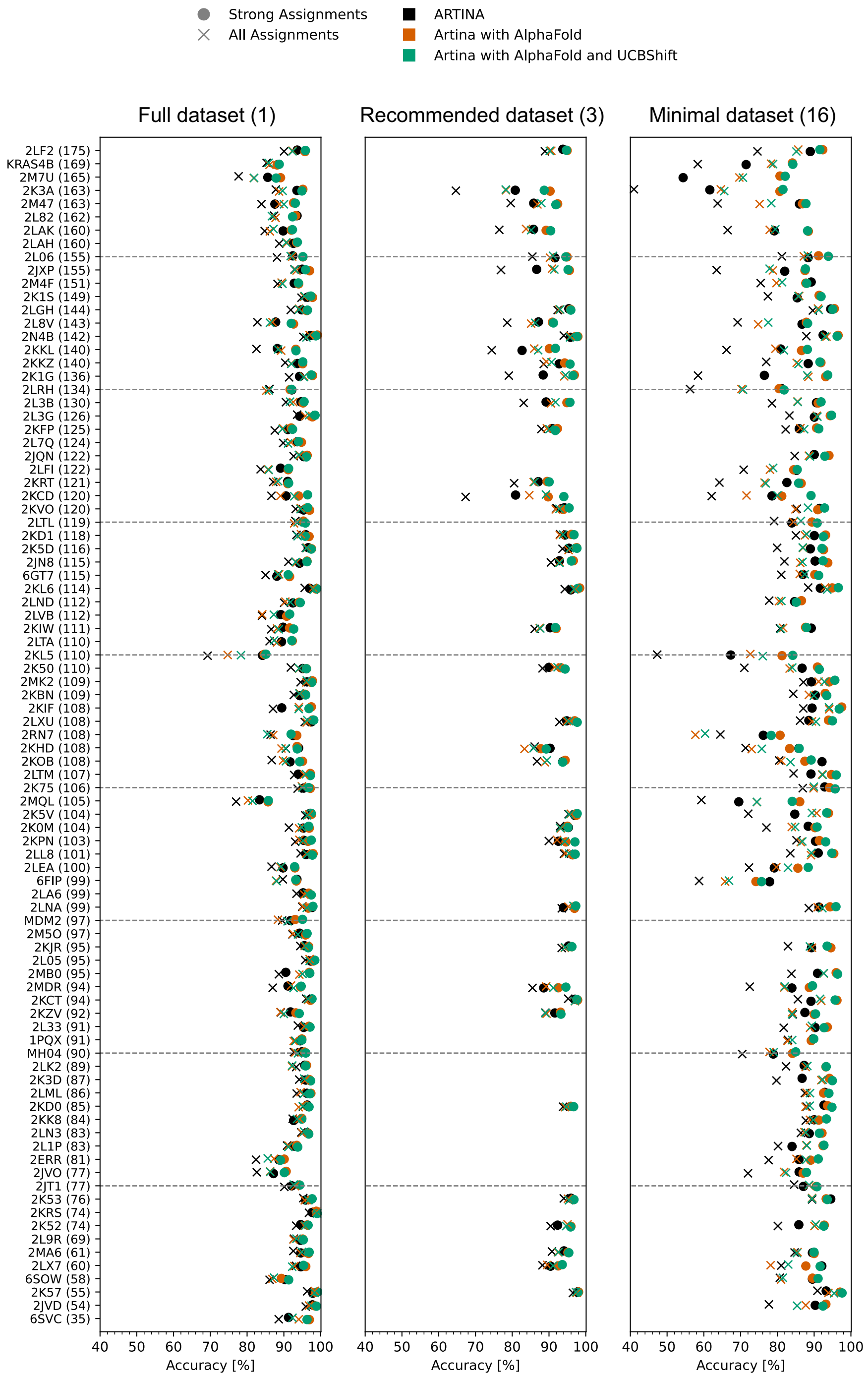

Figure S5. Relation between chemical shift assignment accuracy and protein size. The accuracy of chemical shift assignment is depicted across six experimental settings for each protein with experimental data available in the ARTINA NMR spectra dataset. Protein codes (vertical axis) are sorted by the sequence length, enabling an evaluation of system performance dependency on the size of the analyzed system. In the experiments utilizing the full spectra set (dataset 1), the method demonstrates robustness irrespective of the analyzed system size. Using the recommended spectra set (dataset 3) without any complementary inputs (AlphaFold, UCBShift), a performance decline is observable for certain proteins with a sequence length 120 residues or more (e.g., 2K3A, 2JXP). The inclusion of AlphaFold structures and, optionally, UCBShift predictions mitigates this issue. Further reduction of the input experimental data in the minimal spectra set (dataset 16) amplifies this effect.

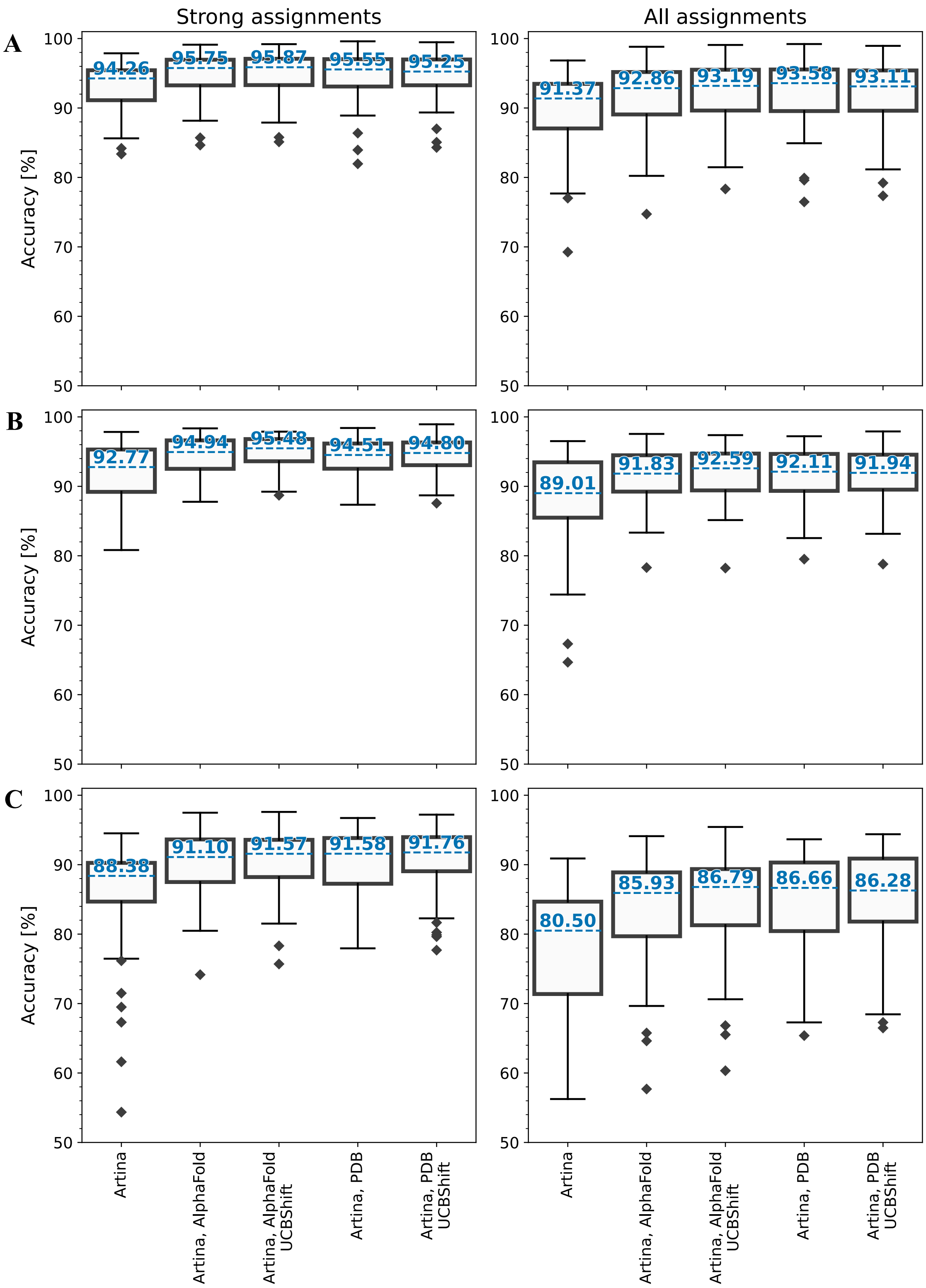

Figure S6. Comparison of assignment accuracy with either deposited PDB or AlphaFold input structures for three spectra subsets. (**A**) Full spectra set (dataset 1). (**B**) Recommended spectra set (dataset 3), (**C**) Minimal spectra set (dataset 16).

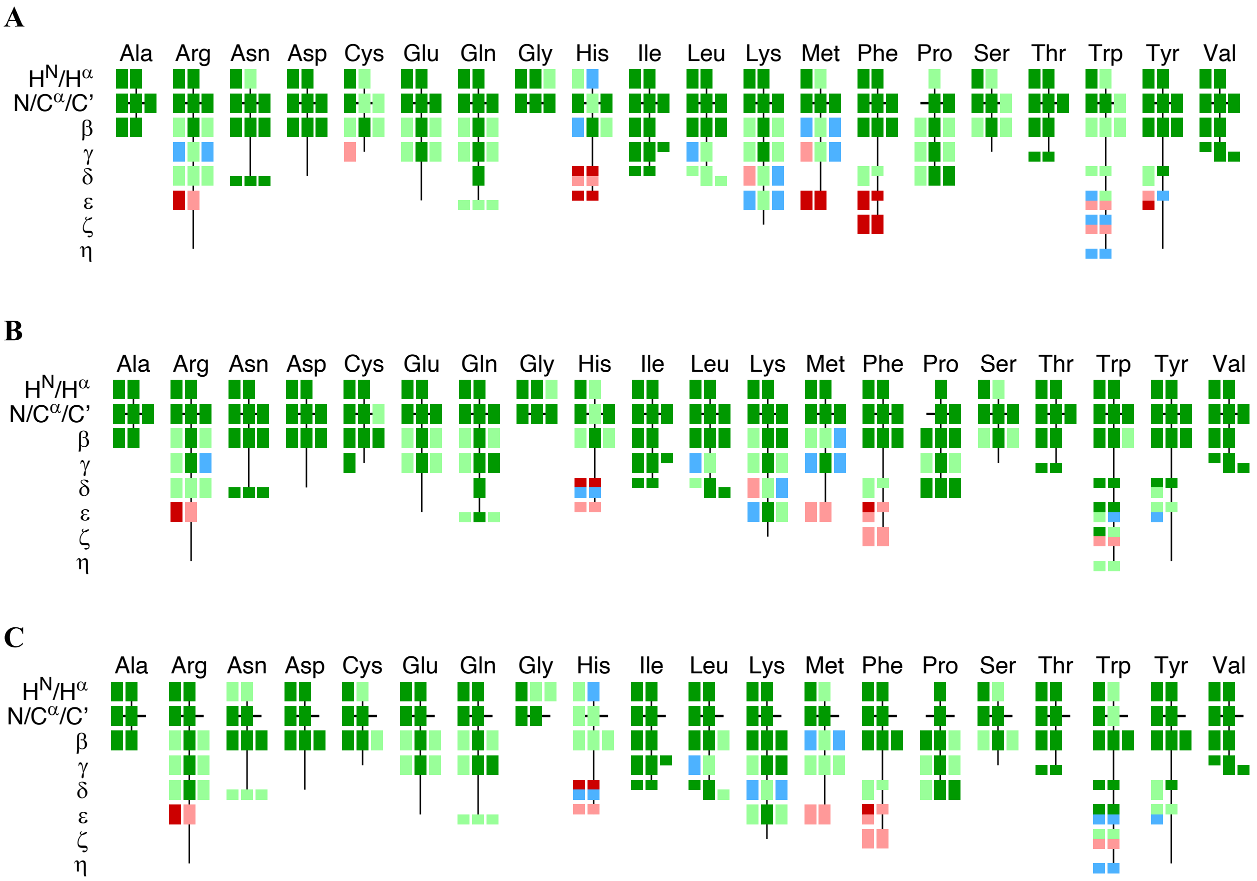

Figure S7. Assignment accuracy for all atom types in the 20 standard amino acids. (**A**) Full spectra set (dataset 1) without structure. (**B**) Full spectra set (dataset 1) with AlphaFold structure and UCBShift predictions. (**C**) Recommended spectra set (dataset 3) with AlphaFold structure and UCBShift predictions. Each atom type in an amino acid is represented by a rectangle colored according to the assignment accuracy 90–100% (dark green), 80–90% (light green), 70–80% (light blue), 50–70% (light red), and 0–50% (dark red). The row labeled H^N/H^α shows for each residue H^N on the left, H^α in the center, and $H^{\alpha3}$ in Gly to the right. The N/C^α/C' row shows for each residue the N, C^α, and C' assignments from left to right. The rows β-η show the side chain assignments for the heavy atoms in the center and hydrogen atoms to the left and right. For branched side chains, the corresponding row is split into an upper part for one branch and a lower part for the other branch.

Table S1. Accuracy of AlphaFold protein structure prediction for 89 benchmark proteins used in this study. The AlphaFold predictions having backbone RMSD to PDB reference above 2 Å have been highlighted in blue. Overall, the average accuracy of AlphaFold on this benchmark dataset is 1.03 Å and 1.58 Å for the backbone and heavy atoms respectively. RMSDs have been calculated for the structured regions of the reference structures listed in Supplementary Table S4 of (Klukowski et al. 2022).

Protein name	Sequence length	AlphaFold backbone RMSD to reference	AlphaFold heavy atom RMSD to reference
6SVC	35	1.25	1.74
2JVD	54	0.32	0.94
2K57	55	0.48	1.22
6SOW	58	0.70	1.43
2LX7	60	2.26	2.81
2MA6	61	0.70	1.17
2L9R	69	0.39	1.03
2K52	74	0.95	1.54
2KRS	74	0.72	1.09
2K53	76	0.57	1.08
2JT1	77	0.54	1.05
2JVO	77	1.41	1.99
2ERR	81	1.51	2.43
2L1P	83	1.40	2.19
2LN3	83	0.54	1.13
2KK8	84	0.96	1.41
2KD0	85	0.81	1.25
2LML	86	0.57	1.25
2K3D	87	1.07	1.75
2LK2	89	0.74	1.42
MH04	90	1.12	1.83
1PQX	91	1.09	1.65
2L33	91	0.61	1.06
2KZV	92	1.24	1.76
2KCT	94	0.40	0.96
2MDR	94	1.32	2.24
2MB0	95	0.63	1.16
2L05	95	0.58	1.19
2KJR	95	1.28	1.80
2M5O	97	0.57	1.01
MDM2	97	1.30	1.90
2LNA	99	0.59	1.16
2LA6	99	0.43	0.94
6FIP	99	2.13	2.42
2LEA	100	1.13	1.76
2LL8	101	0.70	1.10
2KPN	103	0.57	0.87
2K0M	104	0.77	1.19
2K5V	104	0.48	0.98
2MQL	105	0.78	1.21
2K75	106	0.92	1.31
2LTM	107	0.44	0.89
2KOB	108	0.56	0.98
2KHD	108	1.06	1.54
2RN7	108	0.71	1.35
2LXU	108	0.62	1.23
2KIF	108	1.04	1.33
2KBN	109	0.72	1.39
2MK2	109	1.22	1.82
2K50	110	0.55	1.17
2KL5	110	1.31	1.88
2LTA	110	0.98	1.78
2KIW	111	1.29	1.75
2LVB	112	2.66	2.94
2LND	112	2.73	2.96
2KL6	114	0.62	1.16
6GT7	115	0.92	1.55
2JN8	115	1.38	1.94
2K5D	116	1.02	1.46
2KD1	118	0.58	1.23
2LTL	119	0.74	1.06
2KVO	120	1.87	2.50
2KCD	120	1.38	2.02
2KRT	121	1.86	2.69
2LFI	122	1.66	2.08
2JQN	122	1.36	1.81
2L7Q	124	1.03	1.67
2KFP	125	1.42	2.30
2L3G	126	0.43	0.95
2L3B	130	0.76	1.14
2LRH	134	1.57	2.00
2K1G	136	0.53	1.25
2KKZ	140	0.72	1.32
2KKL	140	1.43	1.83
2N4B	142	0.99	1.67
2L8V	143	1.29	2.16
2LGH	144	0.70	1.14
2K1S	149	1.03	1.26
2M4F	151	0.73	1.34
2JXP	155	1.48	2.06
2L06	155	1.08	1.56
2LAH	160	1.01	1.56
2LAK	160	0.73	1.16
2L82	162	1.86	2.30
2M47	163	1.93	2.43
2K3A	163	0.69	1.07
2M7U	165	1.49	2.13
KRAS4B	169	1.25	1.80
2LF2	175	1.88	2.51

Table S2. Chemical shift assignment accuracy (all shifts) calculated independently for each atom type for the full spectra set (dataset 1).

Chemical shift	ARTINA			ARTINA with AlphaFold			ARTINA with AlphaFold and UCBShift		
	Correct	Incorrect	Accuracy (%)	Correct	Incorrect	Accuracy (%)	Correct	Incorrect	Accuracy (%)
**ALA**									
N	561	15	97.40	564	12	97.92	567	9	98.44
C	446	21	95.50	448	19	95.93	449	16	96.56
CA	574	12	97.95	574	12	97.95	572	14	97.61
CB	577	10	98.30	583	4	99.32	583	4	99.32
H	562	16	97.23	567	11	98.1	568	10	98.27
HA	565	20	96.58	565	20	96.58	567	18	96.92
HB	568	18	96.93	576	10	98.29	575	11	98.12
**ARG**									
N	463	17	96.46	473	7	98.54	470	11	97.71
NE	17	12	58.62	17	12	58.62	15	9	62.5
C	343	28	92.45	345	26	92.99	347	24	93.53
CA	466	22	95.49	473	15	96.93	472	16	96.72
CB	449	37	92.39	453	33	93.21	460	26	94.65
CG	411	58	87.63	418	51	89.13	423	46	90.19
CD	416	53	88.7	423	46	90.19	422	47	89.98
H	467	17	96.49	475	9	98.14	471	13	97.31
HA	452	40	91.87	459	33	93.29	462	30	93.9
HB2	403	87	82.24	407	83	83.06	405	85	82.65
HB3	416	71	85.42	420	67	86.24	419	68	86.04
HG2	366	99	78.71	381	84	81.94	377	88	81.08
HG3	351	112	75.81	364	99	78.62	362	101	78.19
HD2	381	81	82.47	405	57	87.66	403	59	87.23
HD3	383	80	82.72	400	63	86.39	401	62	86.61
HE	81	125	39.32	79	127	38.35	74	132	35.92
**ASN**									
N	363	20	94.78	365	18	95.3	369	14	96.34
ND2	347	30	92.04	349	28	92.57	352	25	93.37
C	288	25	92.01	286	26	91.67	290	22	92.95
CA	379	16	95.95	379	16	95.95	383	12	96.96
CB	386	12	96.98	384	14	96.48	387	11	97.24
H	365	22	94.32	366	21	94.57	371	16	95.87
HA	352	42	89.34	357	37	90.61	362	32	91.88
HB2	367	27	93.15	368	26	93.4	371	23	94.16
HB3	365	30	92.41	368	27	93.16	369	26	93.42
HD21	349	29	92.33	348	30	92.06	345	33	91.27
HD22	343	35	90.74	345	33	91.27	346	32	91.53
**ASP**									
N	541	20	96.43	544	17	96.97	546	15	97.33
C	400	38	91.32	408	29	93.36	414	24	94.52
CA	539	23	95.91	549	13	97.69	549	13	97.69
CB	549	18	96.83	553	14	97.53	552	15	97.35
H	539	24	95.74	545	18	96.8	545	18	96.8
HA	525	43	92.43	526	42	92.61	524	44	92.25
HB2	527	40	92.95	532	35	93.83	534	33	94.18
HB3	530	38	93.31	529	39	93.13	531	37	93.49
**CYS**									
N	63	3	95.45	60	6	90.91	63	3	95.45
C	43	5	89.58	42	6	87.5	44	5	89.8
CA	60	7	89.55	59	8	88.06	62	5	92.54
CB	62	6	91.18	62	6	91.18	63	5	92.65
H	63	3	95.45	60	6	90.91	63	3	95.45
HA	59	9	86.76	61	7	89.71	63	5	92.65
HB2	58	10	85.29	60	8	88.24	62	6	91.18
HB3	59	9	86.76	63	5	92.65	62	6	91.18
HG	2	1	66.67	3	0	100	3	0	100

Chemical shift	ARTINA			ARTINA with AlphaFold			ARTINA with AlphaFold and UCBShift		
	Correct	Incorrect	Accuracy (%)	Correct	Incorrect	Accuracy (%)	Correct	Incorrect	Accuracy (%)
**GLU**									
N	760	21	97.31	761	20	97.44	767	14	98.21
C	565	43	92.93	568	40	93.42	570	39	93.6
CA	765	26	96.71	765	26	96.71	771	20	97.47
CB	752	40	94.95	764	28	96.46	760	32	95.96
CG	721	67	91.5	741	47	94.04	736	52	93.4
H	757	25	96.8	757	25	96.8	763	19	97.57
HA	736	52	93.4	744	44	94.42	748	40	94.92
HB2	665	115	85.26	678	102	86.92	674	106	86.41
HB3	681	103	86.86	689	95	87.88	692	92	88.27
HG2	624	154	80.21	652	126	83.8	650	128	83.55
HG3	664	119	84.8	687	96	87.74	686	97	87.61
**GLN**									
N	366	3	99.19	364	5	98.64	366	3	99.19
NE2	304	47	86.61	313	38	89.17	316	35	90.03
C	277	15	94.86	277	14	95.19	277	15	94.86
CA	364	14	96.3	363	15	96.03	366	12	96.83
CB	350	26	93.09	356	20	94.68	357	19	94.95
CG	346	25	93.26	351	20	94.61	351	20	94.61
CD	4	0	100	3	0	100	3	0	100
H	362	8	97.84	363	7	98.11	364	6	98.38
HA	353	21	94.39	359	15	95.99	361	13	96.52
HB2	320	51	86.25	330	41	88.95	331	40	89.22
HB3	323	48	87.06	328	43	88.41	329	42	88.68
HG2	312	56	84.78	317	51	86.14	318	50	86.41
HG3	331	40	89.22	340	31	91.64	338	33	91.11
HE21	308	45	87.25	318	35	90.08	316	37	89.52
HE22	310	41	88.32	306	45	87.18	311	40	88.6
**GLY**									
N	569	21	96.44	567	23	96.1	576	14	97.63
C	437	20	95.62	442	15	96.72	439	17	96.27
CA	590	14	97.68	588	16	97.35	593	11	98.18
H	577	15	97.47	578	14	97.64	575	17	97.13
HA2	547	53	91.17	541	59	90.17	543	57	90.5
HA3	539	62	89.68	531	70	88.35	533	68	88.69
**HIS**									
N	152	16	90.48	155	13	92.26	161	7	95.83
ND1	0	20	0	0	22	0	0	23	0
C	96	8	92.31	94	10	90.38	98	6	94.23
CA	151	27	84.83	149	29	83.71	149	29	83.71
CB	164	17	90.61	164	17	90.61	163	18	90.06
CD2	64	38	62.75	81	23	77.88	80	21	79.21
CE1	21	36	36.84	34	24	58.62	37	21	63.79
H	154	18	89.53	154	18	89.53	160	12	93.02
HA	139	39	78.09	145	33	81.46	145	33	81.46
HB2	140	39	78.21	144	35	80.45	145	34	81.01
HB3	146	33	81.56	150	29	83.8	150	29	83.8
HD1	0	6	0	0	6	0	0	6	0
HD2	75	47	61.48	91	31	74.59	94	28	77.05
HE1	16	55	22.54	38	33	53.52	39	32	54.93

Chemical shift	ARTINA			ARTINA with AlphaFold			ARTINA with AlphaFold and UCBShift		
	Correct	Incorrect	Accuracy (%)	Correct	Incorrect	Accuracy (%)	Correct	Incorrect	Accuracy (%)
**ILE**									
N	545	9	98.38	547	7	98.74	546	8	98.56
C	414	21	95.17	414	21	95.17	418	17	96.09
CA	547	11	98.03	543	15	97.31	544	14	97.49
CB	556	4	99.29	556	4	99.29	555	5	99.11
CG1	539	16	97.12	541	14	97.48	539	16	97.12
CG2	534	24	95.7	536	22	96.06	539	19	96.59
CD1	526	34	93.93	540	20	96.43	538	22	96.07
H	542	12	97.83	545	9	98.38	544	10	98.19
HA	541	16	97.13	539	18	96.77	539	18	96.77
HB	551	8	98.57	547	12	97.85	546	13	97.67
HG12	509	40	92.71	501	48	91.26	503	46	91.62
HG13	518	33	94.01	505	46	91.65	505	46	91.65
HG2	530	27	95.15	531	26	95.33	530	27	95.15
HD1	525	35	93.75	536	24	95.71	535	25	95.54
**LEU**									
N	761	15	98.07	762	15	98.07	762	15	98.07
C	571	34	94.38	577	26	95.69	575	27	95.51
CA	761	24	96.94	770	15	98.09	770	14	98.21
CB	762	23	97.07	770	15	98.09	772	13	98.34
CG	644	117	84.63	650	111	85.41	652	109	85.68
CD1	672	107	86.26	713	67	91.41	703	77	90.13
CD2	670	105	86.45	692	83	89.29	702	73	90.58
H	764	15	98.07	763	16	97.95	762	17	97.82
HA	752	29	96.29	764	17	97.82	764	17	97.82
HB2	709	71	90.9	722	58	92.56	725	55	92.95
HB3	707	72	90.76	718	61	92.17	712	67	91.4
HG	599	165	78.4	599	165	78.4	600	164	78.53
HD1	666	113	85.49	705	74	90.5	698	81	89.6
HD2	677	99	87.24	702	74	90.46	710	66	91.49
**LYS**									
N	637	14	97.85	635	16	97.54	637	14	97.85
C	488	24	95.31	489	23	95.51	491	21	95.9
CA	640	22	96.68	643	19	97.13	648	14	97.89
CB	640	21	96.82	641	20	96.97	645	16	97.58
CG	604	55	91.65	615	44	93.32	618	41	93.78
CD	546	102	84.26	560	88	86.42	555	93	85.65
CE	560	77	87.91	588	49	92.31	589	48	92.46
H	637	15	97.7	634	18	97.24	637	15	97.7
HA	627	34	94.86	635	26	96.07	635	26	96.07
HB2	584	76	88.48	582	78	88.18	584	76	88.48
HB3	580	78	88.15	598	60	90.88	600	58	91.19
HG2	525	126	80.65	539	112	82.8	551	100	84.64
HG3	521	130	80.03	556	95	85.41	549	102	84.33
HD2	392	242	61.83	422	212	66.56	414	220	65.3
HD3	453	183	71.23	465	171	73.11	467	169	73.43
HE2	466	152	75.4	488	130	78.96	487	131	78.8
HE3	494	128	79.42	511	111	82.15	524	98	84.24

Chemical shift	ARTINA			ARTINA with AlphaFold			ARTINA with AlphaFold and UCBShift		
	Correct	Incorrect	Accuracy (%)	Correct	Incorrect	Accuracy (%)	Correct	Incorrect	Accuracy (%)
**MET**									
N	147	7	95.45	147	7	95.45	148	6	96.1
C	114	12	90.48	117	9	92.86	117	9	92.86
CA	166	13	92.74	169	10	94.41	172	7	96.09
CB	157	22	87.71	157	22	87.71	157	22	87.71
CG	150	23	86.71	154	19	89.02	157	16	90.75
CE	58	80	42.03	91	47	65.94	92	46	66.67
H	147	7	95.45	148	6	96.1	147	7	95.45
HA	160	19	89.39	162	17	90.5	165	14	92.18
HB2	141	38	78.77	141	38	78.77	146	33	81.56
HB3	130	47	73.45	133	44	75.14	129	48	72.88
HG2	121	53	69.54	132	42	75.86	132	42	75.86
HG3	133	43	75.57	132	44	75	136	40	77.27
HE	48	90	34.78	89	49	64.49	92	46	66.67
**PHE**									
N	347	17	95.33	358	6	98.35	357	7	98.08
C	257	19	93.12	259	16	94.18	263	13	95.29
CA	349	24	93.57	358	15	95.98	360	13	96.51
CB	355	21	94.41	362	14	96.28	364	12	96.81
CD1	271	55	83.13	280	46	85.89	277	49	84.97
CE1	119	173	40.75	159	132	54.64	158	134	54.11
CZ	70	162	30.17	114	118	49.14	118	114	50.86
H	348	17	95.34	358	7	98.08	354	11	96.99
HA	343	30	91.96	355	18	95.17	357	16	95.71
HB2	343	31	91.71	352	22	94.12	355	19	94.92
HB3	347	28	92.53	354	21	94.4	357	18	95.2
HD1	305	58	84.02	313	50	86.23	313	50	86.23
HD2	289	70	80.5	296	63	82.45	298	61	83.01
HE1	91	230	28.35	158	163	49.22	157	164	48.91
HE2	107	212	33.54	167	152	52.35	178	141	55.8
HZ	67	183	26.8	125	125	50	129	121	51.6
**PRO**									
C	233	18	92.83	234	16	93.6	240	11	95.62
CA	315	26	92.38	327	14	95.89	328	13	96.19
CB	321	17	94.97	326	12	96.45	329	9	97.34
CG	314	24	92.9	317	21	93.79	321	17	94.97
CD	317	23	93.24	325	15	95.59	323	17	95
HA	305	34	89.97	318	21	93.81	320	19	94.4
HB2	296	41	87.83	304	33	90.21	306	31	90.8
HB3	301	38	88.79	305	34	89.97	308	31	90.86
HG2	279	60	82.3	291	48	85.84	290	49	85.55
HG3	281	55	83.63	289	47	86.01	289	47	86.01
HD2	293	42	87.46	302	33	90.15	304	31	90.75
HD3	306	30	91.07	315	21	93.75	315	21	93.75
**SER**									
N	534	24	95.7	535	23	95.88	540	18	96.77
C	414	47	89.8	425	36	92.19	429	32	93.06
CA	532	43	92.52	534	41	92.87	545	30	94.78
CB	535	34	94.02	539	30	94.73	544	25	95.61
H	535	26	95.37	533	28	95.01	541	20	96.43
HA	504	72	87.5	500	76	86.81	515	61	89.41
HB2	491	74	86.9	500	65	88.5	503	62	89.03
HB3	485	83	85.39	484	84	85.21	491	77	86.44

Chemical shift	ARTINA			ARTINA with AlphaFold			ARTINA with AlphaFold and UCBShift		
	Correct	Incorrect	Accuracy (%)	Correct	Incorrect	Accuracy (%)	Correct	Incorrect	Accuracy (%)
**ILE**									
N	545	9	98.38	547	7	98.74	546	8	98.56
C	414	21	95.17	414	21	95.17	418	17	96.09
CA	547	11	98.03	543	15	97.31	544	14	97.49
CB	556	4	99.29	556	4	99.29	555	5	99.11
CG1	539	16	97.12	541	14	97.48	539	16	97.12
CG2	534	24	95.7	536	22	96.06	539	19	96.59
CD1	526	34	93.93	540	20	96.43	538	22	96.07
H	542	12	97.83	545	9	98.38	544	10	98.19
HA	541	16	97.13	539	18	96.77	539	18	96.77
HB	551	8	98.57	547	12	97.85	546	13	97.67
HG12	509	40	92.71	501	48	91.26	503	46	91.62
HG13	518	33	94.01	505	46	91.65	505	46	91.65
HG2	530	27	95.15	531	26	95.33	530	27	95.15
HD1	525	35	93.75	536	24	95.71	535	25	95.54
**LEU**									
N	761	15	98.07	762	15	98.07	762	15	98.07
C	571	34	94.38	577	26	95.69	575	27	95.51
CA	761	24	96.94	770	15	98.09	770	14	98.21
CB	762	23	97.07	770	15	98.09	772	13	98.34
CG	644	117	84.63	650	111	85.41	652	109	85.68
CD1	672	107	86.26	713	67	91.41	703	77	90.13
CD2	670	105	86.45	692	83	89.29	702	73	90.58
H	764	15	98.07	763	16	97.95	762	17	97.82
HA	752	29	96.29	764	17	97.82	764	17	97.82
HB2	709	71	90.9	722	58	92.56	725	55	92.95
HB3	707	72	90.76	718	61	92.17	712	67	91.4
HG	599	165	78.4	599	165	78.4	600	164	78.53
HD1	666	113	85.49	705	74	90.5	698	81	89.6
HD2	677	99	87.24	702	74	90.46	710	66	91.49
**LYS**									
N	637	14	97.85	635	16	97.54	637	14	97.85
C	488	24	95.31	489	23	95.51	491	21	95.9
CA	640	22	96.68	643	19	97.13	648	14	97.89
CB	640	21	96.82	641	20	96.97	645	16	97.58
CG	604	55	91.65	615	44	93.32	618	41	93.78
CD	546	102	84.26	560	88	86.42	555	93	85.65
CE	560	77	87.91	588	49	92.31	589	48	92.46
H	637	15	97.7	634	18	97.24	637	15	97.7
HA	627	34	94.86	635	26	96.07	635	26	96.07
HB2	584	76	88.48	582	78	88.18	584	76	88.48
HB3	580	78	88.15	598	60	90.88	600	58	91.19
HG2	525	126	80.65	539	112	82.8	551	100	84.64
HG3	521	130	80.03	556	95	85.41	549	102	84.33
HD2	392	242	61.83	422	212	66.56	414	220	65.3
HD3	453	183	71.23	465	171	73.11	467	169	73.43
HE2	466	152	75.4	488	130	78.96	487	131	78.8
HE3	494	128	79.42	511	111	82.15	524	98	84.24

Chemical shift	ARTINA			ARTINA with AlphaFold			ARTINA with AlphaFold and UCBShift		
	Correct	Incorrect	Accuracy (%)	Correct	Incorrect	Accuracy (%)	Correct	Incorrect	Accuracy (%)
**MET**									
N	147	7	95.45	147	7	95.45	148	6	96.1
C	114	12	90.48	117	9	92.86	117	9	92.86
CA	166	13	92.74	169	10	94.41	172	7	96.09
CB	157	22	87.71	157	22	87.71	157	22	87.71
CG	150	23	86.71	154	19	89.02	157	16	90.75
CE	58	80	42.03	91	47	65.94	92	46	66.67
H	147	7	95.45	148	6	96.1	147	7	95.45
HA	160	19	89.39	162	17	90.5	165	14	92.18
HB2	141	38	78.77	141	38	78.77	146	33	81.56
HB3	130	47	73.45	133	44	75.14	129	48	72.88
HG2	121	53	69.54	132	42	75.86	132	42	75.86
HG3	133	43	75.57	132	44	75	136	40	77.27
HE	48	90	34.78	89	49	64.49	92	46	66.67
**PHE**									
N	347	17	95.33	358	6	98.35	357	7	98.08
C	257	19	93.12	259	16	94.18	263	13	95.29
CA	349	24	93.57	358	15	95.98	360	13	96.51
CB	355	21	94.41	362	14	96.28	364	12	96.81
CD1	271	55	83.13	280	46	85.89	277	49	84.97
CE1	119	173	40.75	159	132	54.64	158	134	54.11
CZ	70	162	30.17	114	118	49.14	118	114	50.86
H	348	17	95.34	358	7	98.08	354	11	96.99
HA	343	30	91.96	355	18	95.17	357	16	95.71
HB2	343	31	91.71	352	22	94.12	355	19	94.92
HB3	347	28	92.53	354	21	94.4	357	18	95.2
HD1	305	58	84.02	313	50	86.23	313	50	86.23
HD2	289	70	80.5	296	63	82.45	298	61	83.01
HE1	91	230	28.35	158	163	49.22	157	164	48.91
HE2	107	212	33.54	167	152	52.35	178	141	55.8
HZ	67	183	26.8	125	125	50	129	121	51.6
**PRO**									
C	233	18	92.83	234	16	93.6	240	11	95.62
CA	315	26	92.38	327	14	95.89	328	13	96.19
CB	321	17	94.97	326	12	96.45	329	9	97.34
CG	314	24	92.9	317	21	93.79	321	17	94.97
CD	317	23	93.24	325	15	95.59	323	17	95
HA	305	34	89.97	318	21	93.81	320	19	94.4
HB2	296	41	87.83	304	33	90.21	306	31	90.8
HB3	301	38	88.79	305	34	89.97	308	31	90.86
HG2	279	60	82.3	291	48	85.84	290	49	85.55
HG3	281	55	83.63	289	47	86.01	289	47	86.01
HD2	293	42	87.46	302	33	90.15	304	31	90.75
HD3	306	30	91.07	315	21	93.75	315	21	93.75
**SER**									
N	534	24	95.7	535	23	95.88	540	18	96.77
C	414	47	89.8	425	36	92.19	429	32	93.06
CA	532	43	92.52	534	41	92.87	545	30	94.78
CB	535	34	94.02	539	30	94.73	544	25	95.61
H	535	26	95.37	533	28	95.01	541	20	96.43
HA	504	72	87.5	500	76	86.81	515	61	89.41
HB2	491	74	86.9	500	65	88.5	503	62	89.03
HB3	485	83	85.39	484	84	85.21	491	77	86.44

Chemical shift	ARTINA			ARTINA with AlphaFold			ARTINA with AlphaFold and UCBShift		
	Correct	Incorrect	Accuracy (%)	Correct	Incorrect	Accuracy (%)	Correct	Incorrect	Accuracy (%)
**THR**									
N	460	8	98.29	460	8	98.29	458	10	97.86
C	383	17	95.75	383	18	95.51	384	17	95.76
CA	475	13	97.34	469	19	96.11	474	14	97.13
CB	470	17	96.51	470	17	96.51	469	18	96.3
CG2	472	14	97.12	468	18	96.3	471	15	96.91
H	458	13	97.24	459	12	97.45	457	14	97.03
HA	444	45	90.8	442	47	90.39	442	47	90.39
HB	447	35	92.74	450	32	93.36	448	34	92.95
HG2	470	17	96.51	470	17	96.51	472	15	96.92
**TRP**									
N	102	8	92.73	105	5	95.45	105	5	95.45
NE1	90	17	84.11	101	6	94.39	101	6	94.39
C	85	13	86.73	89	9	90.82	91	7	92.86
CA	102	11	90.27	103	10	91.15	106	7	93.81
CB	99	14	87.61	103	10	91.15	105	8	92.92
CD1	89	21	80.91	101	9	91.82	101	9	91.82
CE3	53	33	61.63	64	22	74.42	64	23	73.56
CH2	78	28	73.58	85	22	79.44	89	18	83.18
CZ2	85	23	78.7	97	12	88.99	97	12	88.99
CZ3	50	47	51.55	57	41	58.16	57	41	58.16
H	103	7	93.64	106	4	96.36	106	4	96.36
HA	97	15	86.61	101	11	90.18	102	10	91.07
HB2	97	16	85.84	103	10	91.15	104	9	92.04
HB3	94	18	83.93	98	14	87.5	99	13	88.39
HD1	92	21	81.42	105	8	92.92	103	10	91.15
HE1	87	26	76.99	101	12	89.38	102	11	90.27
HE3	57	34	62.64	72	19	79.12	73	18	80.22
HH2	78	30	72.22	85	23	78.7	90	18	83.33
HZ2	86	26	76.79	99	13	88.39	102	10	91.07
HZ3	59	42	58.42	68	33	67.33	66	35	65.35
**TYR**									
N	272	9	96.8	278	3	98.93	274	7	97.51
C	190	12	94.06	194	8	96.04	191	11	94.55
CA	266	21	92.68	268	19	93.38	272	15	94.77
CB	271	16	94.43	278	9	96.86	280	7	97.56
CD1	234	21	91.76	237	17	93.31	238	16	93.7
CE1	182	68	72.8	214	38	84.92	213	39	84.52
H	272	9	96.8	277	4	98.58	273	8	97.15
HA	261	26	90.94	270	17	94.08	271	16	94.43
HB2	260	26	90.91	270	16	94.41	263	23	91.96
HB3	266	21	92.68	274	13	95.47	273	14	95.12
HD1	241	40	85.77	253	28	90.04	254	27	90.39
HD2	238	40	85.61	242	36	87.05	241	36	87
HE1	167	105	61.4	227	45	83.46	227	45	83.46
HE2	131	138	48.7	197	72	73.23	198	71	73.61
**VAL**									
N	664	18	97.36	669	13	98.09	665	17	97.51
C	504	27	94.92	503	27	94.91	505	25	95.28
CA	671	12	98.24	667	16	97.66	667	16	97.66
CB	675	12	98.25	674	13	98.11	676	11	98.4
CG1	653	30	95.61	647	36	94.73	645	38	94.44
CG2	641	39	94.26	640	40	94.12	642	38	94.41
H	667	16	97.66	669	14	97.95	666	17	97.51
HA	668	16	97.66	664	20	97.08	662	22	96.78
HB	676	10	98.54	672	14	97.96	674	12	98.25
HG1	659	25	96.35	653	31	95.47	654	30	95.61
HG2	648	35	94.88	652	31	95.46	650	33	95.17

Table S3. Chemical shift assignment accuracy (all shifts) calculated independently for each atom type for the recommended spectra set (dataset 3).

Chemical shift	ARTINA			ARTINA with AlphaFold			ARTINA with AlphaFold and UCBShift		
	Correct	Incorrect	Accuracy (%)	Correct	Incorrect	Accuracy (%)	Correct	Incorrect	Accuracy (%)
**ALA**									
N	288	27	91.43	302	13	95.87	301	14	95.6
CA	303	20	93.81	314	9	97.21	316	7	97.8
CB	307	16	95.05	314	9	97.21	315	8	97.5
H	291	26	91.80	304	13	95.90	305	12	96.2
HA	297	26	91.95	307	16	95.05	308	15	95.4
HB	301	22	93.19	312	11	96.59	311	12	96.3
**ARG**									
N	207	21	90.79	215	13	94.30	218	10	95.6
NE	10	12	45.45	13	5	72.22	10	6	62.5
CA	204	26	88.70	218	12	94.78	222	8	96.5
CB	197	29	87.17	201	25	88.94	208	18	92
CG	185	33	84.86	195	23	89.45	198	20	90.8
CD	200	19	91.32	204	15	93.15	203	16	92.7
H	207	23	90.00	219	11	95.22	219	11	95.2
HA	199	31	86.52	214	16	93.04	215	15	93.5
HB2	185	43	81.14	192	36	84.21	192	36	84.2
HB3	188	40	82.46	196	32	85.96	199	29	87.3
HG2	168	51	76.71	176	43	80.37	182	37	83.1
HG3	172	47	78.54	172	47	78.54	188	31	85.8
HD2	179	39	82.11	193	25	88.53	190	28	87.2
HD3	184	33	84.79	195	22	89.86	195	22	89.9
HE	35	70	33.33	39	66	37.14	32	73	30.5
**ASN**									
N	160	23	87.43	162	21	88.52	165	18	90.2
ND2	160	22	87.91	161	21	88.46	159	23	87.4
CA	167	22	88.36	180	9	95.24	182	7	96.3
CB	168	20	89.36	177	11	94.15	182	6	96.8
H	160	25	86.49	162	23	87.57	166	19	89.7
HA	158	30	84.04	165	23	87.77	169	19	89.9
HB2	163	24	87.17	172	15	91.98	176	11	94.1
HB3	167	20	89.30	173	14	92.51	175	12	93.6
HD21	158	23	87.29	159	22	87.85	159	22	87.9
HD22	159	22	87.85	162	19	89.50	159	22	87.9
**ASP**									
N	246	16	93.89	252	10	96.18	254	8	97
CA	251	15	94.36	253	13	95.11	258	8	97
CB	254	12	95.49	256	10	96.24	259	7	97.4
H	247	18	93.21	252	13	95.09	254	11	95.9
HA	241	25	90.60	242	24	90.98	246	20	92.5
HB2	246	20	92.48	250	16	93.98	255	11	95.9
HB3	247	19	92.86	249	17	93.61	247	19	92.9
**CYS**									
N	37	1	97.37	35	3	92.11	35	3	92.1
CA	31	7	81.58	37	1	97.37	36	2	94.7
CB	29	9	76.32	34	4	89.47	35	3	92.1
H	37	1	97.37	36	2	94.74	35	3	92.1
HA	32	6	84.21	34	4	89.47	34	4	89.5
HB2	29	9	76.32	34	4	89.47	36	2	94.7
HB3	31	7	81.58	35	3	92.11	34	4	89.5

Chemical shift	ARTINA			ARTINA with AlphaFold			ARTINA with AlphaFold and UCBShift		
	Correct	Incorrect	Accuracy (%)	Correct	Incorrect	Accuracy (%)	Correct	Incorrect	Accuracy (%)
**GLU**									
N	350	26	93.09	362	14	96.28	362	14	96.3
CA	348	33	91.34	363	18	95.28	371	10	97.4
CB	344	37	90.29	355	26	93.18	357	24	93.7
CG	339	40	89.45	349	30	92.08	354	25	93.4
H	352	24	93.62	362	14	96.28	363	13	96.5
HA	343	38	90.03	356	25	93.44	363	18	95.3
HB2	308	73	80.84	317	64	83.20	327	54	85.8
HB3	316	65	82.94	329	52	86.35	338	43	88.7
HG2	294	86	77.37	300	80	78.95	316	64	83.2
HG3	316	64	83.16	338	42	88.95	341	39	89.7
**GLN**									
N	135	15	90.00	146	4	97.33	146	4	97.3
NE2	113	25	81.88	118	20	85.51	121	17	87.7
CA	141	13	91.56	146	8	94.81	148	6	96.1
CB	136	18	88.31	146	8	94.81	149	5	96.8
CG	138	13	91.39	141	10	93.38	146	5	96.7
H	134	16	89.33	145	5	96.67	146	4	97.3
HA	140	13	91.50	147	6	96.08	147	6	96.1
HB2	127	26	83.01	128	25	83.66	133	20	86.9
HB3	124	29	81.05	132	21	86.27	132	21	86.3
HG2	129	22	85.43	132	19	87.42	134	17	88.7
HG3	127	24	84.11	130	21	86.09	137	14	90.7
HE21	114	26	81.43	120	20	85.71	124	16	88.6
HE22	114	25	82.01	117	22	84.17	117	22	84.2
**GLY**									
N	293	25	92.14	297	21	93.40	303	15	95.3
CA	301	20	93.77	308	13	95.95	310	11	96.6
H	299	20	93.73	299	20	93.73	303	16	95
HA2	281	39	87.81	282	38	88.13	276	44	86.3
HA3	267	53	83.44	278	42	86.88	281	39	87.8
**HIS**									
N	79	14	84.95	82	11	88.17	81	12	87.1
ND1	0	19	0.00	0	19	0.00	0	20	0
CA	76	23	76.77	81	18	81.82	82	17	82.8
CB	83	16	83.84	86	13	86.87	88	11	88.9
CD2	33	19	63.46	36	16	69.23	38	14	73.1
CE1	11	27	28.95	23	16	58.97	24	14	63.2
H	79	15	84.04	83	11	88.30	80	14	85.1
HA	67	30	69.07	75	22	77.32	76	21	78.4
HB2	73	24	75.26	81	16	83.51	82	15	84.5
HB3	76	21	78.35	80	17	82.47	80	17	82.5
HD1	0	4	0.00	0	4	0.00	0	4	0
HD2	33	25	56.90	42	16	72.41	42	16	72.4
HE1	7	33	17.50	23	17	57.50	24	16	60

Chemical shift	ARTINA			ARTINA with AlphaFold			ARTINA with AlphaFold and UCBShift		
	Correct	Incorrect	Accuracy (%)	Correct	Incorrect	Accuracy (%)	Correct	Incorrect	Accuracy (%)
**ILE**									
N	237	18	92.94	249	6	97.65	247	8	96.9
CA	245	13	94.96	253	5	98.06	254	4	98.5
CB	249	10	96.14	254	5	98.07	255	4	98.5
CG1	247	11	95.74	254	4	98.45	254	4	98.5
CG2	243	16	93.82	250	9	96.53	250	9	96.5
CD1	241	18	93.05	250	9	96.53	249	10	96.1
H	236	19	92.55	247	8	96.86	247	8	96.9
HA	244	15	94.21	250	9	96.53	250	9	96.5
HB	247	12	95.37	250	9	96.53	250	9	96.5
HG12	235	22	91.44	236	21	91.83	237	20	92.2
HG13	234	23	91.05	238	19	92.61	239	18	93
HG2	245	14	94.59	249	10	96.14	248	11	95.8
HD1	241	18	93.05	249	10	96.14	248	11	95.8
**LEU**									
N	339	22	93.91	350	11	96.95	353	8	97.8
CA	346	23	93.77	366	3	99.19	365	4	98.9
CB	342	27	92.68	366	3	99.19	365	4	98.9
CG	308	57	84.38	305	60	83.56	305	60	83.6
CD1	317	51	86.14	343	25	93.21	343	25	93.2
CD2	317	52	85.91	331	38	89.70	336	33	91.1
H	341	22	93.94	353	10	97.25	353	10	97.3
HA	339	30	91.87	363	6	98.37	361	8	97.8
HB2	320	48	86.96	345	23	93.75	345	23	93.8
HB3	313	54	85.29	329	38	89.65	327	40	89.1
HG	267	97	73.35	271	93	74.45	274	90	75.3
HD1	306	62	83.15	333	35	90.49	337	31	91.6
HD2	316	53	85.64	332	37	89.97	332	37	90
**LYS**									
N	279	15	94.90	279	15	94.90	283	11	96.3
CA	293	7	97.67	293	7	97.67	293	7	97.7
CB	288	10	96.64	290	8	97.32	292	6	98
CG	276	19	93.56	278	17	94.24	277	18	93.9
CD	249	44	84.98	260	33	88.74	259	34	88.4
CE	267	24	91.75	271	20	93.13	271	20	93.1
H	281	13	95.58	279	15	94.90	284	10	96.6
HA	287	14	95.35	292	9	97.01	292	9	97
HB2	276	23	92.31	270	29	90.30	274	25	91.6
HB3	261	36	87.88	271	26	91.25	274	23	92.3
HG2	249	45	84.69	250	44	85.03	254	40	86.4
HG3	251	43	85.37	258	36	87.76	265	29	90.1
HD2	187	106	63.82	207	86	70.65	207	86	70.7
HD3	212	80	72.60	222	70	76.03	221	71	75.7
HE2	228	52	81.43	231	49	82.50	229	51	81.8
HE3	231	49	82.50	237	43	84.64	237	43	84.6

Chemical shift	ARTINA			ARTINA with AlphaFold			ARTINA with AlphaFold and UCBShift		
	Correct	Incorrect	Accuracy (%)	Correct	Incorrect	Accuracy (%)	Correct	Incorrect	Accuracy (%)
**MET**									
N	64	7	90.14	68	3	95.77	69	2	97.2
CA	74	14	84.09	76	12	86.36	80	8	90.9
CB	67	18	78.82	68	17	80.00	69	16	81.2
CG	65	18	78.31	71	12	85.54	74	9	89.2
CE	31	38	44.93	45	24	65.22	45	23	66.2
H	64	7	90.14	69	2	97.18	69	2	97.2
HA	73	14	83.91	73	14	83.91	75	12	86.2
HB2	58	26	69.05	64	20	76.19	67	17	79.8
HB3	59	25	70.24	62	22	73.81	61	23	72.6
HG2	58	26	69.05	66	18	78.57	69	15	82.1
HG3	58	26	69.05	64	20	76.19	68	16	81
HE	22	47	31.88	42	27	60.87	43	26	62.3
PHE									
N	169	14	92.35	178	5	97.27	178	5	97.3
CA	165	22	88.24	179	8	95.72	182	5	97.3
CB	169	20	89.42	179	10	94.71	182	7	96.3
CD1	145	35	80.56	158	22	87.78	156	24	86.7
CE1	66	88	42.86	78	75	50.98	82	71	53.6
CZ	39	71	35.45	56	54	50.91	57	53	51.8
H	169	15	91.85	178	6	96.74	177	7	96.2
HA	163	25	86.70	176	12	93.62	179	9	95.2
HB2	164	25	86.77	176	13	93.12	179	10	94.7
HB3	158	31	83.60	174	15	92.06	176	13	93.1
HD1	151	34	81.62	160	25	86.49	161	24	87
HD2	148	37	80.00	163	22	88.11	164	21	88.7
HE1	45	116	27.95	79	82	49.07	79	82	49.1
HE2	48	113	29.81	90	71	55.90	82	79	50.9
HZ	33	85	27.97	66	52	55.93	68	50	57.6
**PRO**									
CA	158	19	89.27	170	7	96.05	172	5	97.2
CB	162	15	91.53	167	10	94.35	170	7	96.1
CG	159	17	90.34	165	11	93.75	168	8	95.5
CD	163	12	93.14	169	6	96.57	170	5	97.1
HA	152	25	85.88	166	11	93.79	168	9	94.9
HB2	151	26	85.31	158	19	89.27	161	16	91
HB3	151	26	85.31	152	25	85.88	156	21	88.1
HG2	137	39	77.84	144	32	81.82	145	31	82.4
HG3	144	32	81.82	151	25	85.80	152	24	86.4
HD2	146	29	83.43	151	24	86.29	155	20	88.6
HD3	149	26	85.14	165	10	94.29	165	10	94.3
**SER**									
N	244	29	89.38	252	21	92.31	254	19	93
CA	239	39	85.97	260	18	93.53	265	13	95.3
CB	241	35	87.32	261	15	94.57	263	13	95.3
H	245	29	89.42	254	20	92.70	255	19	93.1
HA	223	55	80.22	239	39	85.97	245	33	88.1
HB2	218	60	78.42	234	44	84.17	237	41	85.3
HB3	215	62	77.62	229	48	82.67	231	46	83.4

Chemical shift	ARTINA			ARTINA with AlphaFold			ARTINA with AlphaFold and UCBShift		
	Correct	Incorrect	Accuracy (%)	Correct	Incorrect	Accuracy (%)	Correct	Incorrect	Accuracy (%)
**THR**									
N	227	18	92.65	232	13	94.69	230	15	93.9
CA	229	25	90.16	243	11	95.67	247	7	97.2
CB	230	23	90.91	247	6	97.63	243	10	96.1
CG2	236	14	94.40	242	8	96.80	244	6	97.6
H	223	25	89.92	233	15	93.95	230	18	92.7
HA	217	37	85.43	225	29	88.58	230	24	90.6
HB	218	35	86.17	230	23	90.91	230	23	90.9
HG2	232	19	92.43	241	10	96.02	240	11	95.6
**TRP**									
N	60	5	92.31	60	5	92.31	62	3	95.4
NE1	53	7	88.33	58	2	96.67	58	2	96.7
CA	58	8	87.88	57	9	86.36	59	7	89.4
CB	58	8	87.88	58	8	87.88	61	5	92.4
CD1	54	9	85.71	59	4	93.65	59	4	93.7
CE3	26	20	56.52	34	12	73.91	35	11	76.1
CH2	42	19	68.85	46	15	75.41	48	13	78.7
CZ2	55	9	85.94	55	9	85.94	57	7	89.1
CZ3	29	24	54.72	28	25	52.83	28	25	52.8
H	61	4	93.85	61	4	93.85	62	3	95.4
HA	56	10	84.85	57	9	86.36	58	8	87.9
HB2	55	11	83.33	59	7	89.39	62	4	93.9
HB3	56	10	84.85	57	9	86.36	61	5	92.4
HD1	54	11	83.08	62	3	95.38	59	6	90.8
HE1	52	13	80.00	59	6	90.77	59	6	90.8
HE3	28	20	58.33	35	13	72.92	36	12	75
HH2	42	19	68.85	45	16	73.77	47	14	77.1
HZ2	52	12	81.25	55	9	85.94	55	9	85.9
HZ3	35	20	63.64	35	20	63.64	37	18	67.3
**TYR**									
N	130	12	91.55	140	2	98.59	141	1	99.3
CA	131	15	89.73	139	7	95.21	140	6	95.9
CB	128	17	88.28	138	7	95.17	139	6	95.9
CD1	114	21	84.44	123	12	91.11	122	12	91
CE1	93	37	71.54	113	17	86.92	110	20	84.6
H	131	11	92.25	139	3	97.89	140	2	98.6
HA	127	19	86.99	132	14	90.41	133	13	91.1
HB2	124	21	85.52	131	14	90.34	132	13	91
HB3	126	19	86.90	132	13	91.03	135	10	93.1
HD1	117	24	82.98	125	16	88.65	126	15	89.4
HD2	113	28	80.14	125	16	88.65	125	16	88.7
HE1	81	49	62.31	108	22	83.08	110	20	84.6
HE2	66	64	50.77	103	27	79.23	104	26	80
**VAL**									
N	306	25	92.45	320	11	96.68	323	8	97.6
CA	321	16	95.25	336	1	99.70	336	1	99.7
CB	319	18	94.66	332	5	98.52	333	4	98.8
CG1	316	21	93.77	325	12	96.44	323	14	95.9
CG2	313	21	93.71	320	14	95.81	321	13	96.1
H	308	24	92.77	322	10	96.99	321	11	96.7
HA	319	18	94.66	329	8	97.63	330	7	97.9
HB	321	16	95.25	333	4	98.81	333	4	98.8
HG1	314	23	93.18	332	5	98.52	326	11	96.7
HG2	317	17	94.91	328	6	98.20	327	7	97.9

Table S4. Chemical shift assignments for all shifts within and outside well-structured regions for the full spectra set (dataset 1).

Protein	Sequence length	ARTINA				ARTINA with AlphaFold				ARTINA with AlphaFold and UCBShift			
		Structured		Disordered		Structured		Disordered		Structured		Disordered	
		Correct	Incorrect	Correct	Incorrect	Correct	Incorrect	Correct	Incorrect	Correct	Incorrect	Correct	Incorrect
6SVC	35	298	27	13	13	315	11	17	10	310	17	16	10
2JVD	54	392	15	153	8	397	10	153	8	400	7	154	7
2K57	55	567	23	38	0	577	10	38	0	578	10	38	0
6SOW	58	534	39	49	55	541	33	48	56	538	37	55	49
2LX7	60	605	43	22	2	601	47	23	1	599	50	22	2
2MA6	61	531	27	92	23	534	24	102	13	531	27	108	7
2L9R	69	533	32	145	21	533	33	145	21	537	29	147	19
2K52	74	769	53	46	5	786	36	49	2	790	32	47	4
2KRS	74	664	22	72	2	677	9	74	0	679	7	74	0
2K53	76	497	18	189	16	499	16	190	15	499	16	197	8
2JT1	77	614	61	98	17	634	42	102	13	634	43	100	15
2JVO	77	593	126	21	3	620	99	21	3	620	99	21	3
2ERR	81	598	130	21	2	634	94	22	1	621	107	22	1
2L1P	83	723	72	44	5	728	67	42	7	729	66	41	8
2LN3	83	822	38	43	10	827	33	40	13	831	29	41	12
2KK8	84	778	64	0	0	794	48	0	0	788	54	0	0
2KD0	85	789	40	40	2	787	42	32	10	794	35	35	7
2LML	86	832	60	42	1	843	50	42	1	851	42	42	1
2K3D	87	881	50	9	5	895	36	10	4	894	37	9	5
2LK2	89	615	35	257	27	606	44	255	29	606	44	256	28
MH04	90	922	72	23	2	926	68	23	2	933	61	23	2
1PQX	91	766	48	135	20	761	53	137	18	765	49	138	17
2L33	91	551	30	236	22	569	12	232	26	570	11	236	22
2KZV	92	748	97	109	7	756	89	100	16	765	80	100	16
2KCT	94	750	23	176	15	755	18	176	15	753	20	178	13
2MDR	94	707	114	61	1	751	70	61	1	758	63	60	2
2MB0	95	647	72	181	34	683	36	197	18	690	29	198	17
2L05	95	831	34	129	8	843	22	129	8	842	23	130	7
2KJR	95	838	50	34	1	848	39	34	1	851	36	34	1
2M5O	97	800	49	81	24	802	47	80	25	809	40	85	20
MDM2	97	796	100	61	0	785	111	61	0	810	86	61	0
2LNA	99	798	40	187	12	805	33	182	17	805	33	193	6
2LA6	99	900	55	26	10	912	43	35	1	921	34	34	2
6FIP	99	959	107	54	9	944	122	48	15	942	124	52	11
2LEA	100	585	76	200	45	597	64	210	35	596	65	213	32
2LL8	101	946	43	61	15	963	26	60	16	965	24	61	15
2KPN	103	806	42	163	30	811	36	169	24	824	23	166	27
2K0M	104	847	87	138	6	880	54	135	9	888	46	135	9
2K5V	104	899	38	180	9	916	21	175	14	911	26	174	15
2MQL	105	541	144	203	78	578	107	197	84	584	101	203	78
2K75	106	958	67	90	3	972	53	89	4	973	51	89	4
2LTM	107	999	66	35	14	1026	39	38	11	1032	33	38	11
2KOB	108	947	143	70	13	976	115	78	5	985	106	78	5
2KHD	108	687	72	283	29	699	60	258	54	702	57	267	45
2RN7	108	493	35	364	100	498	30	366	98	491	37	357	107

Protein	Sequence length	ARTINA				ARTINA with AlphaFold				ARTINA with AlphaFold and UCBShift			
		Structured		Disordered		Structured		Disordered		Structured		Disordered	
		Correct	Incorrect	Correct	Incorrect	Correct	Incorrect	Correct	Incorrect	Correct	Incorrect	Correct	Incorrect
2LXU	108	994	43	138	8	1001	36	137	9	1001	36	139	7
2KIF	108	957	138	18	7	1031	64	23	2	1030	65	23	2
2KBN	109	983	82	124	5	1001	63	125	4	999	65	125	4
2MK2	109	1013	52	12	7	1026	39	16	3	1028	37	15	4
2K50	110	911	61	189	36	934	38	193	32	936	36	191	34
2KL5	110	626	254	7	27	664	216	19	15	693	187	23	11
2LTA	110	1027	154	58	21	1043	138	67	11	1028	153	72	6
2KIW	111	914	134	183	36	945	103	172	47	941	105	180	39
2LVB	112	952	166	38	22	952	166	39	21	982	135	45	15
2LND	112	1001	104	61	13	1009	96	57	17	1023	82	59	15
2KL6	114	1143	48	28	5	1168	23	31	2	1169	22	32	1
6GT7	115	802	127	73	27	842	87	71	29	836	93	74	26
2JN8	115	974	85	140	22	996	62	144	18	993	65	144	18
2K5D	116	825	31	370	19	833	23	366	23	830	26	367	22
2KD1	118	1105	82	119	4	1122	65	118	5	1111	76	117	6
2LTL	119	965	62	119	18	952	75	128	9	962	65	129	8
2KVO	120	1051	79	146	8	1070	60	145	9	1070	60	144	10
2KCD	120	1110	178	56	2	1141	146	57	1	1193	93	55	3
2KRT	121	1060	159	41	4	1069	150	42	3	1077	142	41	4
2LFI	122	966	194	116	17	991	169	117	16	993	166	117	16
2JQN	122	1139	86	34	7	1165	61	31	10	1159	67	33	8
2L7Q	124	960	103	265	36	984	80	272	30	974	90	271	31
2KFP	125	1174	167	25	4	1203	138	27	2	1203	138	25	4
2L3G	126	1184	83	48	1	1212	55	49	0	1226	41	49	0
2L3B	130	971	80	248	47	992	58	253	42	980	72	255	40
2LRH	134	1313	210	62	12	1296	226	64	10	1306	216	65	9
2K1G	136	1227	114	106	11	1280	62	114	3	1277	64	114	3
2KKZ	140	1187	113	202	35	1204	96	210	27	1208	92	207	30
2KKL	140	789	208	411	46	889	109	410	47	877	122	411	46
2N4B	142	1202	48	259	24	1217	33	270	13	1206	44	268	15
2L8V	143	1026	163	38	58	1063	126	54	42	1055	134	53	43
2LGH	144	1320	116	39	4	1355	81	37	6	1352	84	40	3
2K1S	149	1416	78	44	2	1429	66	43	3	1431	63	42	4
2M4F	151	1043	102	430	93	1067	79	425	98	1073	73	423	100
2JXP	155	1404	105	113	11	1412	97	114	10	1405	104	114	10
2L06	155	1228	182	214	12	1291	118	218	8	1286	121	214	12
2LAH	160	1365	172	85	12	1402	136	84	13	1393	144	88	9
2LAK	160	867	95	489	148	889	75	491	146	899	65	497	140
2L82	162	1467	208	40	10	1474	200	39	11	1457	217	39	11
2M47	163	1164	198	224	68	1231	131	237	55	1237	124	251	41
2K3A	163	821	75	548	115	841	55	541	121	839	56	557	105
2M7U	165	1189	276	47	79	1254	210	47	79	1253	213	51	75
KRAS4B	169	1260	206	182	42	1265	201	190	34	1266	200	185	39
2LF2	175	1445	157	275	33	1477	125	287	21	1478	124	286	22
**Accuracy**		**90.63**		**85.15**		**92.53**		**86.00**		**92.71**		**86.84**	

Table S5. Chemical shift assignments for all shifts within and outside well-structured regions for the recommended spectra set (dataset 3).

Protein	Sequence length	ARTINA				ARTINA with AlphaFold				ARTINA with AlphaFold and UCBShift			
		Structured		Disordered		Structured		Disordered		Structured		Disordered	
		Correct	Incorrect	Correct	Incorrect	Correct	Incorrect	Correct	Incorrect	Correct	Incorrect	Correct	Incorrect
2K1G	136	967	259	87	20	1157	68	97	10	1161	64	98	9
2K50	110	797	94	171	35	841	50	163	43	842	49	171	35
2KFP	125	1090	150	24	3	1108	132	25	2	1116	124	26	1
2KJR	95	756	54	30	1	766	44	27	4	764	45	29	2
2KKL	140	649	264	340	76	779	133	363	53	794	119	363	53
2KOB	108	870	131	64	12	891	110	66	10	896	105	67	9
2LX7	60	537	62	10	11	536	63	19	2	544	55	21	0
2MDR	94	694	127	61	1	751	70	37	25	750	71	55	7
2JN8	115	884	84	127	22	902	66	135	14	905	63	132	17
2K3A	163	601	207	305	288	736	72	361	232	744	64	352	241
2K52	74	688	71	41	6	723	36	43	4	720	39	43	4
2K5V	104	819	37	161	11	831	25	155	17	827	29	155	17
2KD0	85	718	40	28	9	722	36	28	9	723	35	30	7
2KHD	108	620	75	222	61	618	77	197	86	634	61	205	78
2KKZ	140	1071	121	178	40	1089	103	170	48	1098	94	181	37
2L06	155	1097	203	193	16	1171	128	190	19	1182	115	190	19
2L3B	130	810	156	219	53	896	70	225	47	906	60	230	42
2L8V	143	888	202	44	51	947	143	62	33	958	132	64	31
2LXU	108	898	55	109	23	909	44	120	12	914	39	121	11
2K5D	116	739	44	325	28	759	24	324	29	759	24	328	25
2KD1	118	1014	77	106	7	1016	75	103	10	1025	65	104	9
2KPN	103	709	61	142	34	730	41	143	33	741	29	151	25
2KRT	121	900	224	38	3	960	164	40	1	964	160	39	2
2KVO	120	950	85	132	8	947	88	132	8	960	75	131	9
2LAK	160	755	126	366	219	791	91	438	147	794	88	455	130
2LF2	175	1302	159	251	35	1321	140	262	24	1313	148	263	23
2LL8	101	866	42	53	16	873	35	50	19	878	30	54	14
2LNA	99	726	44	164	18	738	32	169	13	742	28	178	4
2MA6	61	482	32	82	25	487	27	87	20	488	26	87	20
2JXP	155	1056	318	88	25	1256	118	95	18	1262	112	98	15
2K0M	104	791	67	129	2	800	58	122	9	799	59	121	10
2K57	55	519	20	34	0	527	10	30	4	526	11	30	4
2KCD	120	790	394	42	10	995	189	51	1	1052	132	50	2
2KCT	94	673	31	166	11	687	17	168	9	687	17	168	9
2KL6	114	1034	54	21	10	1056	32	31	0	1053	35	30	1
2KZV	92	684	90	102	7	693	80	95	14	690	84	96	13
2LGH	144	1216	94	31	9	1220	90	33	7	1223	87	33	7
2M47	163	1007	238	198	71	1097	148	218	51	1110	135	221	48
2K53	76	492	23	185	20	496	19	189	16	494	21	193	12
2KIW	111	843	119	161	43	863	99	157	47	862	100	160	44
2N4B	142	1087	58	231	26	1109	36	235	22	1106	39	236	21
**Accuracy (%)**		**87.68**		**79.99**		**92.07**		**82.83**		**92.62**		**84.37**	
